# In Silico Design, Synthesis, and Evaluation of Novel Enantiopure Isoxazolidines as Promising Dual Inhibitors of α-Amylase and α-Glucosidase

**DOI:** 10.3390/molecules29020305

**Published:** 2024-01-06

**Authors:** Fahad Alhawday, Fahad Alminderej, Siwar Ghannay, Bechir Hammami, Abuzar E. A. E. Albadri, Adel Kadri, Kaiss Aouadi

**Affiliations:** 1 Department of Chemistry, College of Science, Qassim University, Buraidah 51452, Saudi Arabia; 431114194@qu.edu.sa (F.A.); f.alminderej@qu.edu.sa (F.A.); s.ghannay@qu.edu.sa (S.G.); b.hammami@qu.edu.sa (B.H.); aa.albadri@qu.edu.sa (A.E.A.E.A.); 2 Faculty of Sciences of Bizerte FSB, University of Carthage, Jarzouna 7021, Tunisia; 3 Department of Chemistry, Faculty of Science of Sfax, University of Sfax, B.P. 1171, Sfax 3000, Tunisia; 4 Faculty of Science and Arts in Baljurashi, Al-Baha University, P.O. Box 1988, Al-Baha 65527, Saudi Arabia; 5 Laboratory of Heterocyclic Chemistry, LR11ES39, Department of Chemistry, Faculty of Science of Monastir, University of Monastir, Avenue of the Environment, Monastir 5019, Tunisia

**Keywords:** diabetes mellitus, molecular docking, α-amylase, α-glucosidase, isoxazolidine, ADMET, dynamic simulation

## Abstract

Isoxazolidine derivatives were designed, synthesized, and characterized using different spectroscopic techniques and elemental analysis and then evaluated for their ability to inhibit both α-amylase and α-glucosidase enzymes to treat diabetes. All synthesized derivatives demonstrated a varying range of activity, with IC_50_ values ranging from 53.03 ± 0.106 to 232.8 ± 0.517 μM (α-amylase) and from 94.33 ± 0.282 to 258.7 ± 0.521 μM (α-glucosidase), revealing their high potency compared to the reference drug, acarbose (IC_50_ = 296.6 ± 0.825 µM and 780.4 ± 0.346 µM), respectively. Specifically, in vitro results revealed that compound **5d** achieved the most inhibitory activity with IC_50_ values of 5.59-fold and 8.27-fold, respectively, toward both enzymes, followed by **5b**. Kinetic studies revealed that compound **5d** inhibits both enzymes in a competitive mode. Based on the structure–activity relationship (SAR) study, it was concluded that various substitution patterns of the substituent(s) influenced the inhibitory activities of both enzymes. The server pkCSM was used to predict the pharmacokinetics and drug-likeness properties for **5d**, which afforded good oral bioavailability. Additionally, compound **5d** was subjected to molecular docking to gain insights into its binding mode interactions with the target enzymes. Moreover, via molecular dynamics (MD) simulation analysis, it maintained stability throughout 100 ns. This suggests that **5d** possesses the potential to simultaneously target both enzymes effectively, making it advantageous for the development of antidiabetic medications.

## 1. Introduction

**Figure 1:**
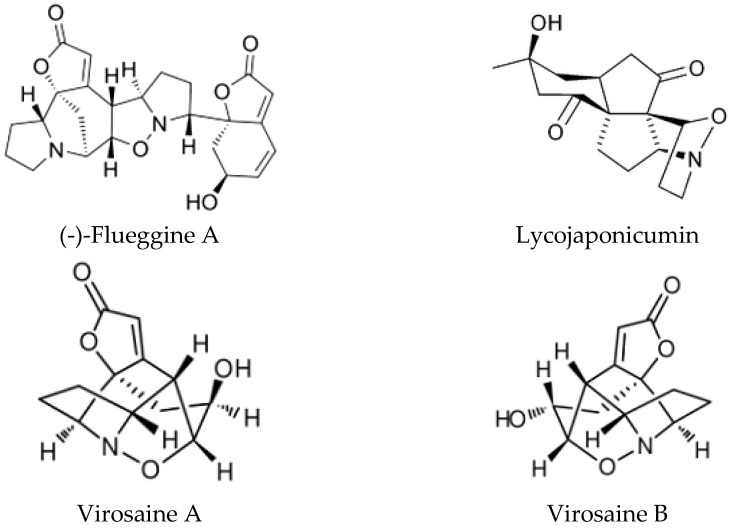
Some natural isoxazolidine derivatives.

The autoimmune and noncommunicable diabetes mellitus (DM) disease, mainly affected by oxidative stress, is a chronic metabolic and endocrine disorder, including Type 1 (T1D), Type 2 (T2D), and gestational diabetes mellitus (GD), which is often characterized by hyperglycemia (due to defects in insulin resistance and insufficient insulin secretion by pancreatic B-cells) [1,2]. DM-induced oxidative stress is substantially associated with Advanced Glycation End Products (AGEs), inducing serious difficulties and a deterioration in life expectancy with a high level of premature mortality, which results in micro- and macrovascular complications (due to neurodegeneration and peripheral cell damage) [3,4,5]. Oxidative stress is characterized by the development of reactive oxygen and nitrogen species (RONS), which progresses by hampering enzymatic and nonenzymatic activities [6,7]. The International Diabetes Federation (IDF) estimated that there will be 578 million cases of diabetes worldwide by 2023, which can reach 643 million in 2030 and 783 million in 2045, of which 1.21% (7 million) were from Saudi Arabia (representing 17.7% of the adult population) [8]. With the high prevalence of T2D induced by an abnormal postprandial increase in blood glucose level, accounting for around 90% of all diabetes, one of the viable prophylactic approaches to managing postprandial hyperglycemia in T2D is reducing carbohydrate digestibility through the inhibition of α-amylase and α-glucosidase [9,10,11,12,13,14,15]. T2D, in which genetic predispositions, overweight, physical inactivity, and other environmental factors work together to generate a variable degree of insulin resistance and dysfunction of β-pancreatic cells, leads to hyperglycemia as the main pathophysiological consequence [16,17]. However, some developed sugar or glycomimetic derivatives have served as anti-T2D, including acarbose, voglibose, and miglitol [18]. Miglitol and voglibose act selectively by blocking the enzyme α-glucosidase, while acarbose plays a dual role by inhibiting α-amylase and α-glucosidase [19]. These drugs represent various side effects, such as liver damage, kidney dysfunction, diarrhea, bloating, flatulence, pain, and abdominal discomfort. Therefore, to cure diabetes and minimize side effects as much as possible, medicinal chemists leaned towards the design, synthesis, and development of new potent α-amylase and *α*-glucosidase inhibitors with small structures, high efficacy, and low side effects. A recent study showed that compounds containing an isoxazolidine ring can be antidiabetic [11,20]. Additionally, some natural isoxazolidine derivatives are known for their diverse biological activities [21,22,23] (Figure 1), such as anticancer [24], antibacterial [25], antifungal [26], and antioxidant [27] properties. Isoxazolidine analogs have also been used as a key intermediate to obtain natural compounds of high biological importance [28,29,30], such as 4-hydroxyisoleucine [31], a natural hypoglycemic agent.

In view of the above and within the framework of our ongoing research on novel antidiabetic agents [11,20], as well as in the design and synthesis of potent heterocyclic derivatives [32,33,34], the aim of the present study is the synthesis of some new isoxazolidine derivatives through 1,3-dipolar cycloaddition. These derivatives are intended as potential inhibitors of α-amylase and *α*-glucosidase, and their inhibitory effects were further supported through in silico investigations, including pharmacokinetics, molecular docking, and dynamic simulations.

## 2. Results

### 2.1. Chemistry

**Scheme 1:**
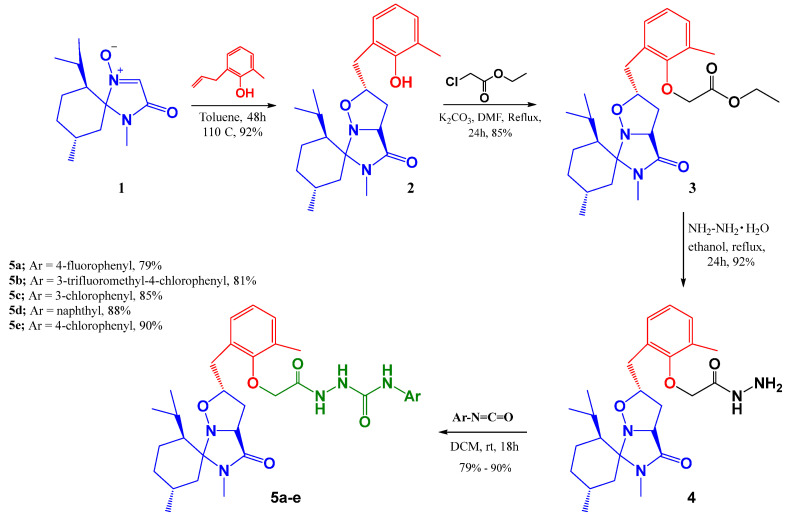
Synthesis of enantiopure isoxazolidine derivatives **5a–e**.

**Scheme 2:**
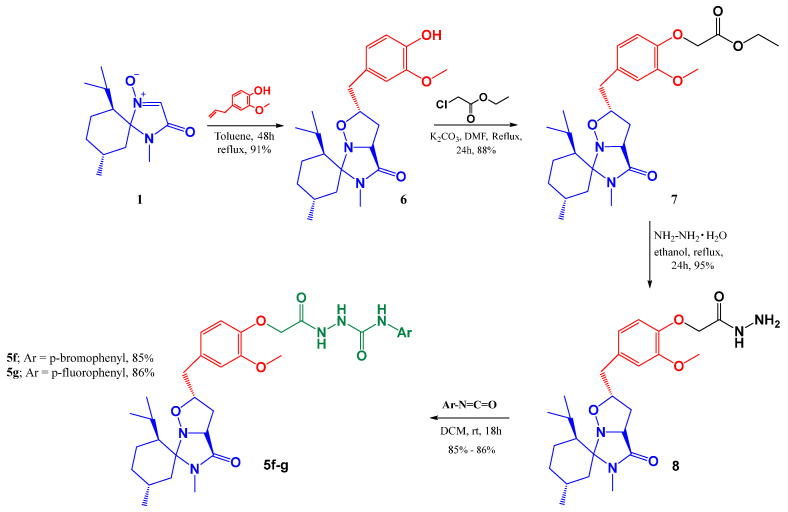
Synthesis of enantiopure semicarbazide derivatives **5f,g**.

2-Allyl-6-methylphenol reacted with the nitrone derived from (-)-menthone [35,36] to yield the cycloadduct **2**. The alkylation of isoxazolidine **2**, in the presence of chloromethyl ethyl ester in a basic medium, produced the intermediate **3**. The condensation of ester **3** with monohydrate hydrazine allowed the formation of hydrazide **4** (Supplementary Materials, pages from 2 to 3). Hydrazide **4** reacted with isocyanate derivatives through a condensation reaction to result in the enantiopure isoxazolidine derivatives **5a–e** (Supplementary Materials, pages from 6 to 15) (Scheme 1). The same reaction sequence was applied using 2-allyl-5-methoxyphenol as a dipolarophile to access compound **8** (Supplementary Materials, pages from 4 to 5). Hydrazide **8** was condensed with *p*-bromophenyl isocyanate and *p*-fluorophenyl isocyanate to yield the semicarbazides **5f** and **5g** (Supplementary Materials, pages from 16 to 19), respectively (Scheme 2).

The stereochemistry of compounds **2** and **6** was determined in a recent work by Alminderej et al. [24]. The structures of the synthesized compounds **5a–g** and the most relevant coupling constants are illustrated in Table 1.

### 2.2. In Vitro α-Amylase and α-Glucosidase Inhibitory Activities 

Both α-amylase and α-glucosidase inhibition have been explored as effective therapeutic approaches against chronic T2D. Therefore, a series of newly synthesized enantiopure isoxazolidine has been assessed for their in vitro enzyme inhibition activity against α-amylase and α-glucosidase, with Acarbose as a standard drug (Table 2). All synthesized analogs exhibited excellent inhibitory potential towards α-amylase and α-glucosidase, with IC_50_ values ranging from 53 ± 0.106 to 232.8 ± 0.517 μM and from 94.33 ± 0.282 to 258.7 ± 0.521 μM, respectively, compared with acarbose (296.6 ± 0.825 μM and 780.4 ± 0.346 μM). Among all the antihyperglycemic agents, compound **5d** was found to be a strong inhibitor of both enzymes, with the highest α-amylase (IC_50_ = 53.03 ± 0.106 µM) and α-glucosidase (IC_50_ = 94.33 ± 0.282 µM) inhibitory activity, which were 5.6 and 8.3-fold higher than that of acarbose. Also, the inhibitory effect of **5b** (IC_50_ = 118.9 ± 0.325 µM) and **5c** (IC_50_ = 120.9 ± 0.333 µM) was not significantly (*p* > 0.05) different towards α-glucosidase, but **5b** (IC_50_ = 67.4 ± 0.202 µM) showed a significantly (*p* < 0.05) different inhibition activity than **5c** (IC_50_ = 92.28 ± 0.276 µM) against α-amylase. Compound **5e** was the least active within the series.

### 2.3. Enzyme Kinetic Studies 

#### 2.3.1. Mode of α-Amylase Inhibition

**Figure 2:**
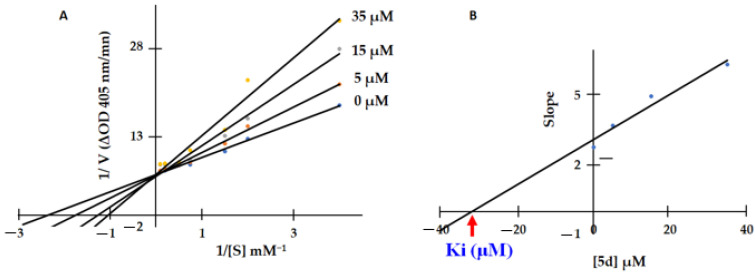
(**A**) Lineweaver–Burk plot of the kinetics of α-amylase inhibition by **5d**. (**B**) Secondary re-plot of Lineweaver–Burk plots between the slopes of each line on the Lineweaver–Burk plot versus various concentrations of **5d**.

The kinetic study of α-amylase for the most potent compound, **5d**, was performed. As shown in Figure 2A, the Lineweaver–Burk graphs of different concentrations of inhibitors revealed that, as the concentrations of inhibitors increased, Vmax values remained unchanged. However, Km values gradually increased, suggesting that compound **5d** was a competitive inhibitor against α-glucosidase. The inhibition constant, Ki, was estimated via the second re-plot of the Lineweaver–Burk plots vs. the different concentrations of the inhibitor (Figure 2B) and was found to be 31.9 μM.

#### 2.3.2. Mode of α-Glucosidase Inhibition

**Figure 3:**
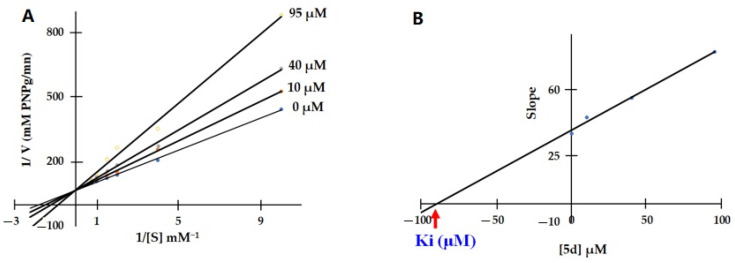
(**A**) Lineweaver–Burk plot of the kinetics of α-glucosidase inhibition by **5d**. (**B**) Secondary re-plot of Lineweaver–Burk plots between the slopes of each line on the Lineweaver–Burk plot versus various concentrations of **5d**.

According to the results depicted in Figure 3A, the Lineweaver–Burk plot showed that the Km increases and Vmax remains essentially constant with increasing inhibitor concentration, indicating that **5d** binds to the active site and inhibits α-glucosidase in a competitive manner. Moreover, an estimated value of Ki was determined by plotting the slope for each straight-line concentration versus different concentrations of **5d**, revealing a value of 89.5 μM (Figure 3B). 

### 2.4. Structure–Activity Relationship (SAR) Investigation 

**Figure 4:**
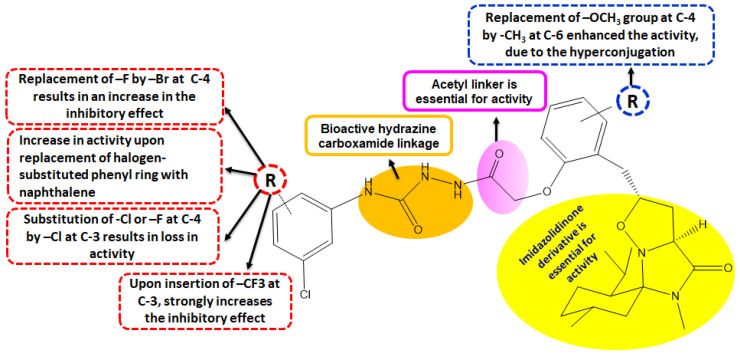
Generalized SAR of the synthesized compounds.

Based on the in vitro α-amylase and α-glucosidase inhibitory effects of the synthesized analogs, a preliminary SAR was considered to indicate the effect of substituents on the enzymatic inhibitory potential of the synthesized analogs and those substituents on the phenyl ring played a critical role in modulating the activity of the compounds. The better activity of **5d** was mainly attributed to the presence of a naphthalene moiety, widely found in natural products with proven biological activities. Additionally, compound **5e**, with *p*-chlorophenyl moiety, showed the lowest inhibitory activity. However, the introduction of a trifluoromethyl (–CF_3_) group (**5b**) on the *p*-chlorophenyl moiety remarkably enhanced the enzymatic activity. In fact, –CF_3_ is believed to improve the pharmacodynamics and pharmacokinetic properties of the resulting compounds, which is confirmed by many drugs having -CF_3_, such as hydroxyflutamide, cinacalcet, and dutasteride [37]. Moreover, a -Cl group in the *meta* position (**5c**, *m*-chlorophenyl) can tremendously increase the enzymatic potency compared to **5e** (*p*-chlorophenyl) but statistically remains lower than that of **5b**. The significant inhibitory effect of compound **5a** was attributed to the presence of an -F group as an electron-withdrawing group on the *p*-position of the phenyl ring compared to **5e**, bearing -Cl group at the *para*-position of the phenyl ring. Upon replacement of the *p*-methoxyphenoxy moiety (**5g**) with an *o*-methylphenoxy core (**5a**), the activity decreased due to the hyperconjugation effect of methyl at the *o*-position of the phenyl ring. When the -F group (**5f**) was replaced by a -Br group (**5g**), the inhibitory enzymatic activity was found to be more potent, suggesting that the presence of a -Br group may allow it to form favorable interactions with the target and induce an antihyperglycemic effect (Figure 4).

### 2.5. ADMET Analysis

To minimize the need for experimental procedures while increasing the chances of success, the leading active synthesized compound, **5d**, underwent ADMET analysis [13,15,38,39,40] to assess its predictability properties and demonstrate its effectiveness and safety as a potent drug candidate (Table 3). Results indicated low values for intestinal absorption (86.437%) and good skin permeability. The predicted blood–brain permeation barrier (BBB permeant), the volume of distributions (VDss, extent of drug distribution), and fraction unbound (a portion of free drug in plasma that may extravasate) are crucial distribution pharmacokinetic drug parameters. As shown, moderate penetration of **5d** through CNS (central nervous system) with intermediate values of VDss suggests that the drug is moderately distributed, with interesting fraction unbound values. For drug metabolism in the liver, cytochrome P450s isoenzymes are important parameters, with the most relevant being CYP2D6 and CYP3A4. The prediction of the toxicity profile of **5d** suggests no toxicity.

### 2.6. Molecular Docking Study

Based on the dual inhibitory effect towards α-amylase and α-glucosidase enzymes exerted by the synthesized compounds, molecular docking studies were performed on two target enzymes (PDB code: 2QV4 and PDB code: 3W37, respectively) to better explore their possible binding pattern in the active site. 

To understand the investigated biological activities by which the synthesized derivatives persuaded their efficacy, the binding affinity, and binding interactions at the active site of the α-glucosidase enzyme and the α-amylase enzymes were investigated. These two enzymes are considered prime targets for antidiabetic drugs, as they play key roles in carbohydrate metabolism, specifically in the breakdown of complex carbohydrates into simpler forms like glucose. By inhibiting the activity of these enzymes, the digestion and absorption of carbohydrates can be slowed down, leading to a decrease in postprandial glucose levels. α-Glucosidase inhibitors work by blocking the action of α-glucosidase in the small intestine, thereby reducing the absorption of glucose. This helps control blood glucose levels after meals and prevents spikes in postprandial glucose. Similarly, α-amylase inhibitors target the α-amylase enzyme, which is responsible for breaking down starches into simpler sugars. Inhibiting α-amylase activity slows down the conversion of dietary starches into glucose, leading to better glucose control. Targeting these enzymes provides a mechanism to regulate postprandial hyperglycemia, a common concern in diabetes management. By developing drugs that selectively inhibit α-glucosidase and α-amylase, it is possible to control the digestion and absorption of carbohydrates, leading to improved glycemic control in individuals with diabetes. The results of the docking run of biologically active synthesized compounds with α-glucosidase and α-amylase are summarized in Table 4.

**Figure 5:**
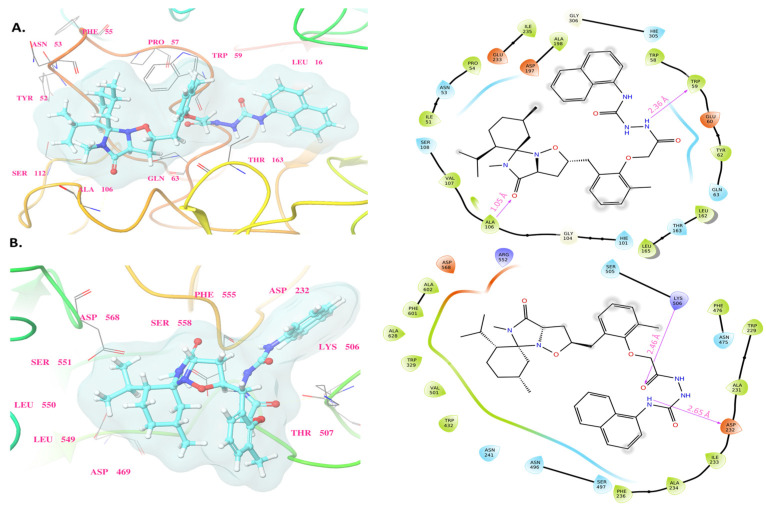
2D and 3D Binding poses of representative compound **5d** in the active site of (**A**) α-amylase enzyme (PDB code: 2QV4) and (**B**) α-glucosidase enzyme (PDB code: 3W37).

For the α-amylase enzyme, **5d** (−5.623 Kcal/mol) demonstrates the highest inhibitory activity among the listed compounds, followed by **5b** (−5.607 Kcal/mol) and **5c** (−5.245 Kcal/mol). Compound **5d** stands out as the most potent inhibitor, indicating its potential to effectively block the action of α-amylase. Regarding the α-glucosidase enzyme, **5c** (−5.55 Kcal/mol) exhibits the strongest inhibitory activity, followed by **5d** (−5.373 Kcal/mol) and **5b** (−5.343 Kcal/mol). Compound **5d** remains the promising inhibitor for this enzyme as well. By considering both enzymes together, **5d** consistently shows the highest inhibitory activity among the listed compounds for both enzymes. This indicates that **5d** has the potential to effectively target both enzymes simultaneously, which is advantageous for developing antidiabetic drugs. The 2D and 3D graphical representations of the **5d**–protein interactions presented in Figure 5 were prepared using Maestro’s ligand-interaction tool. The analysis revealed that **5d** binds to the active binding site of both the α-amylase and α-glucosidase enzymes through weak, noncovalent interactions, predominantly hydrogen bonding and π-cation interactions. In the α-amylase enzyme, **5d** forms two hydrogen bond interactions with crucial amino acid residues Trp59 and Ala106, indicating its affinity for the active site. Similarly, in the α-glucosidase enzyme, **5d** establishes hydrogen bond interactions with Lys506 and Asp232. Notably, the hydrogen bond observed between Asp49 and Acarbose, a known α-glucosidase inhibitor, is also present during the interaction of **5d**, suggesting its potential as an antidiabetic agent.

### 2.7. MD Simulation

**Figure 6:**
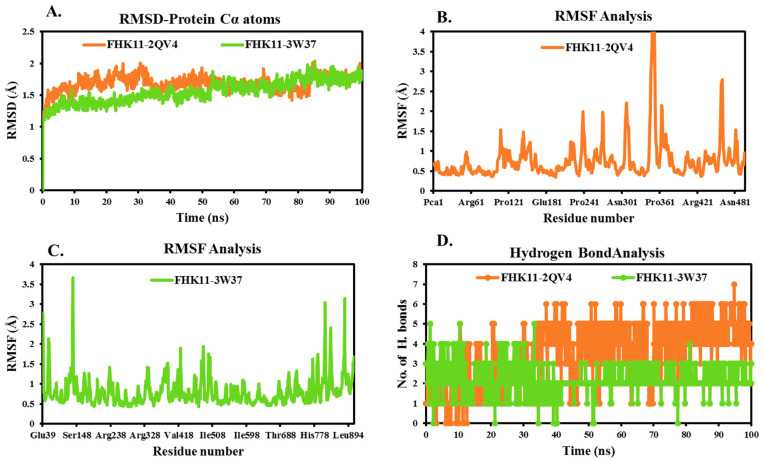
(**A**) Time-dependent RMSD of Cα atoms of the **5d**–2QV4 and **5d**–3W37 complexes; (**B**) RMSF of individual amino acids of Cα atoms of the **5d**–2QV4 complex; (**C**) RMSF of individual amino acids of Cα atoms of the **5d**–3W37 complex; (**D**). Time-dependent Hydrogen bond analysis of the **5d**–2QV4 and **5d**–3W37 complexes.

MD simulations were carried out for 100 ns inside the target binding site of α-amylase (2QV4) and α-glucosidase (3W37) as a function of simulation time to estimate the reactivity and the stability of the synthesized compounds as well as their attitude during their binding to the active site of the proteins [11,20]. Considering both complexes, the RMSD plots of **5d** demonstrate that these complexes quickly reached an equilibrium status and remained stable throughout the simulation after a few nanoseconds. The minimum RMSD values of 1.06 Å and 1.04 Å indicate minimal differences between the reference conformation and the compared conformations (Figure 6A). Furthermore, the average RMSD values of 1.68 Å and 1.55 Å suggest that, on average, **5d**-3W37 exhibits slightly better structural alignment with the reference conformation compared to **5d**-2QV4. Although a slight drift was observed, it remained constant throughout the simulation duration. Importantly, no significant differences were observed in the simulation trajectory of the α-amylase (2QV4) and α-glucosidase (3W37) proteins in the presence of the compound **5d**. The measurement of RMSF is a valuable parameter for gaining an overview of the dynamic behavior of individual residues in the protein backbone depending on their position and participation in the interaction with a given ligand. This approach provides a more comprehensive view of the flexible areas of the protein structure [41,42]. The greater the RMSF value, the greater the atomic variation of the protein’s atomic Cα coordinates from its average location during the MD simulations. As shown in Figure 5, compound **5d** interacted with 24 amino acids of α-amylase protein, including Val49 (0.6 Å), Ile51 (0.7 Å), Pro54 (0.8 Å), Trp58 (0.5 Å), Trp59 (0.6 Å), Tyr62 (0.4 Å), Gln63 (0.4 Å), His101 (0.5 Å), Asn105 (0.8 Å), Ala106 (0.9 Å), Val107 (1.0 Å), Tyr151 (1.0 Å), Arg161 (0.6 Å), Leu162 (0.8 Å), Thr163 (0.9 Å), Gly164 (0.8 Å), Leu165 (0.6 Å), Asp197 (0.6 Å), Ala198 (0.6 Å), Lys200 (0.6 Å), His201 (0.5 Å), His299 (0.6 Å), Asp300 (0.5 Å), and His305 (0.8 Å). All these interacting residues fluctuated in the range of 0.4 to 1 Å. Our results were consistent with those determined previously when studying the X-ray crystal structure of α-amylase (2QV4), suggesting the presence of three main amino acids, namely Asp197, Asp300, and Glu233, which are responsible for its catalytic action (in starch-hydrolyzing enzyme) [43,44,45,46,47,48]. Asp197 was proven to act as a catalytic nucleophile during starch hydrolysis; Glu233 amino acid provides an acid–base catalyst role, while Asp300 plays a leading role in optimizing the orientation of the substrate [49,50]. It is well reported that the redocking of the native ligand (acarbose) to the binding site of α-amylase protein (2 QV4) forms conventional hydrogen bonds with Tyr62, Gln63, Ala106, Val107, Thr163, Gly164, Arg195, His299, Asp300, and Glu233 amino acids, and Van der Waals interactions with Ile51, Trp58, Trp59, Glu60, His101, Gly104, Asn105, Tyr151, Leu162, Leu165, Asp197, Ala198, Lys200, His201, Ile235, Asn298, and His305 [51]. Thus, the binding modes of the formed complex 5d–2QV4 justify its good binding in the acarbose-binding site, sharing many similar residues. While in the **5d**–3W37, 27 amino acids participated in ligand binding, namely Ala231 (0.7 Å), Asp232 (0.8 Å), Ile233 (1.1 Å), Ala234 (1.2 Å), Tyr243 (0.9 Å), Trp329 (0.7 Å), Phe364 (0.6 Å), Ile396 (0.6 Å), Val431 (0.7 Å), Trp432 (0.8 Å), Trp467 (0.6 Å), Met470 (0.7 Å), Ser474 (0.7 Å), Asn475 (0.6 Å), Phe476 (0.9 Å), Ile477 (1.0 Å), Ile503 (1.0 Å), Lys506 (0.8 Å), Arg552 (0.6 Å), Trp565 (0.9 Å), Asp568 (0.7 Å), Phe601 (0.6 Å), Arg629 (0.8 Å), and Asp630 (1.0 Å), These interacting residues have RMSF values in the range of 0.6 to 1.2 Å, indicating that throughout the 100 ns simulation period, the protein–ligand complex remained stable. This limited value of RMSFs revealed that **5d** could develop persistent and appropriate contacts with the protein-binding region during MD simulation. Regarding the binding mode, our results in the case of the **5d**–3W37 complex were found to be in good agreement with the redocked crystal of acarbose with α-glucosidase (3W37) from beet plant, as reported previously [52,53]. In this case, acarbose interacts with 3W37 via hydrogen bonds with the amino acids Asn237, Arg552, and Asp558, as well as by forming salt bridges with Asp232, Asn237, Trp239, Asp469, and Met470, which also matches perfectly with others studies [54,55]. 

These results were completely consistent with the findings of the RMSD plot study. Hydrogen bonding plays a crucial role in determining the stability and functionality of molecular complexes. It is a specific type of intermolecular interaction that occurs between a hydrogen atom bonded to an electronegative atom (donor) and another electronegative atom (acceptor) [56]. These bonds are relatively weak, but they significantly contribute to the overall structure and properties of molecules. In the context of the given analysis, hydrogen bonding analysis provides valuable insights into the interactions between the synthesized compound **5d** and the α-amylase (2QV4) and α-glucosidase (3W37) proteins. The comparison of hydrogen bonding patterns between **5d**–2QV4 and **5d**–3W37 reveals interesting findings when considered alongside the RMSD and RMSF data. The results indicate that **5d**–2QV4 formed a maximum of seven hydrogen bonds. On average, it formed approximately 4.3 hydrogen bonds. On the other hand, **5d**–3W37 formed a maximum of 4 hydrogen bonds, with an average of approximately 2.3 hydrogen bonds. These findings suggest that **5d**–2QV4 had a higher propensity for hydrogen bonding compared to **5d**–3W37. Overall, the combination of RMSD, RMSF, and hydrogen bonding analysis provides a comprehensive understanding of the structural dynamics and stability of the **5d** complexes (Figure 6D). While **5d**–3W37 shows slightly better structural alignment with the reference conformation, **5d**–2QV4 exhibits a higher propensity for forming hydrogen bonds. These observations highlight the complex interplay between hydrogen bonding, structural deviations, and stability, emphasizing the significance of hydrogen bonds in the molecular interactions of **5d** with the α-amylase and α-glucosidase proteins.

## 3. Materials and Methods

### 3.1. Chemistry

#### 3.1.1. General Methods

The solvents used in this study were purchased from Sigma Aldrich. Thin-layer chromatography was carried out on aluminum foils (silica gel 60 F254 (MACHEREY-NAGEL)). Flash silica gel column chromatography was performed with Si 60 silica gel (40–63 µm). NMR spectra were recorded using a 400 MHz spectrometer (Bruker, Buraidah, Saudi Arabia). The chemical shifts are referenced to the residual peaks of deuterated chloroform. HRMS spectra were recorded using a Bruker MicroToF-Q II XL spectrometer (Bruker, Buraidah, Saudi Arabia).

#### 3.1.2. General Procedure (A) for the Preparation of Cycloadduct **2** and **6**

A solution of nitrone (1 eq.) in toluene was combined with eugenol (or 2-allyl-6-methylphenol) (1 eq.). The mixture was refluxed for 48 h with stirring. The obtained cycloadduct was purified by flash chromatography (Cyclohexane/EtOAc 7:3) to separate the desired compound **2** (or **6**).

#### 3.1.3. General Procedure B for the Preparation of Ester **3** and **7**

Compound **2** (or **6**) (1 eq.)**,** ethyl chloroacetate (1.2 eq.), and anhydrous potassium carbonate (1.2 eq.) were refluxed for 24 h in 15 mL of acetone. The resulting mixture was extracted with distilled water and diethyl ether and dried with Na_2_SO_4_. The obtained ester was used in the following reaction without purification.

#### 3.1.4. General Procedure C for the Preparation of Hydrazide **4** and **8**

Hydrazine hydrate (5 mL) was added to ester **3** (or **7**) (500 mg) in ethanol and heated under reflux for 24 h. Ice water was then added to the mixture. The resulting precipitate was filtered with ice water and dried to obtain hydrazide **4** (or **8**).

#### 3.1.5. General Procedure D for the Preparation of Compounds **5a–g**

An equimolecular amount of hydrazide **4** (or **8**) with the appropriate commercial isocyanate was refluxed in ethanol. The reaction mixture was evaporated and dried. The resulting crude residue was purified using flash chromatography (Cyclohexane/EtOAc 7:3) to isolate the desired products **5a–e** (or **5f,g**).

2-(2-(((2S,2′R,3a’R,5R)-2-isopropyl-5,5′-dimethyl-4′-oxotetrahydro-2′H-spiro[cyclohexane-1,6′-imidazo[1,5-b]isoxazol]-2′-yl)methyl)-6-methylphenoxy)acetohydrazide **4**

Compound **4** was obtained according to procedure **C**: hydrazine hydrate (3 eq.), ester **3** (1 eq.). The expected compound was obtained in the form of cotton (95%). 1H NMR (CDCl_3_, 400 MHz) δppm: 0.68 (d, 3H, *J* = 6.4 Hz, CH_3_); 0.80 (d, 3H, *J* = 6.8 Hz, CH_3_); 0.82 (d, 3H, *J* = 6.4 Hz, CH_3_); 0.83–0.87 (m, 1H); 1.11 (t, 1H, *J* = 12.4 Hz); 1.21–1.36 (m, 3H); 1.57–1.77 (m, 5H); 1.88–1.99 (m, 1H); 2.21 (s, 3H, CH_3_); 2.16–2.27 (m, 1H); 2.66 (s, 3H, NCH_3_); 2.63–2.65 (m, 1H); 2.75 (dd, 1H, *J* = 8.4 Hz, 14 Hz); 2.81–2.86 (m, 1H); 3.81–3.85 (m, 2H); 3.96 (d, 1H, *J* = 8.4 Hz); 4.20 (d, 1H, *J* = 14.8 Hz); 4.42 (d, 1H, *J* = 14.8 Hz); 6.95 (t, 1H, *J* = 7.6 Hz); 7.03 (t, 1H, *J* = 7.6 Hz); 8.34 (s, 1H, NH). 13C NMR (CDCl_3_, 100 MHz) δppm: 16.2; 18.2; 21.9; 22.2; 24.0; 24.2; 25.9; 29.5; 32.7; 34.4; 38.9; 40.3; 47.8; 66.1; 70.7; 77.8; 89.8; 124.8; 128.5; 130.0; 130.5; 130.8; 153.9; 168.6; 172.6. Anal. calc. for C_25_H_38_N_4_O_4_ (458.60) (%): C, 65.48; H, 8.35; O, 13.95. Found: C, 65.23; H, 8.44; O, 13.63 HRMS, calcd C_25_H_38_N_4_O_4_ [M + Na]^+^: 481.2791; found 481.2805. 

2-(4-(((2S,2′R,3a’R,5R)-2-isopropyl-5,5′-dimethyl-4′-oxotetrahydro-2′H-spiro[cyclohexane-1,6′-imidazo[1,5-b]isoxazol]-2′-yl)methyl)-2-methoxyphenoxy)acetohydrazide **8**

Compound **8** was obtained according to procedure **C**: hydrazine hydrate (3 eq.), ester **7** (1 eq.). The expected compound was obtained in the form of cotton (95%). 1H NMR (CDCl_3_, 400 MHz) δppm: 0.64 (d, 3H, *J* = 6.4 Hz, CH_3_); 0.78 (d, 3H, *J* = 6.8 Hz, CH_3_); 0.79 (d, 3H, *J* = 6.4 Hz, CH_3_); 0.82 (m, 1H); 1.07 (t, 1H, *J* = 12.4 Hz); 1.20 (t, 1H, *J* = 7.2 Hz); 1.25 (dd, 1H, *J* = 2.8 Hz, 12 Hz); 1.31–1.35 (m, 1H); 1.53–1.66 (m, 3H); 1.71–1.74 (m, 1H); 1.82–1.87 (m, 2H); 2.12–2.19 (m, 1H); 2.60–2.66 (m, 1H); 2.63 (s, 3H, NCH_3_); 2.72–2.75 (m, 2H); 3.73–3.78 (m, 1H); 3.80 (s, 3H, OCH_3_); 3.95 (d, 1H, *J* = 8.4 Hz); 4.51 (s, 2H); 6.68–6.75 (m, 3H); 8.30 (s, 1H, NH). 13C NMR (CDCl_3_, 100 MHz) δppm: 18.2; 21.9; 22.1; 23.9; 24.1; 25.9; 29.6; 34.3; 38.4; 38.6; 40.1; 47.8; 55.6; 66.1; 69.2; 78.3; 89.8; 112.8; 115.3; 121.0; 133.6; 145.5; 149.1; 168.9; 172.5. Anal. calc. for C_25_H_38_N_4_O_5_ (474.60) (%): C, 63.27; H, 8.07; O, 16.86. Found: C, 62.92; H, 7.98; O, 16.77. HRMS, calcd C_25_H_38_N_4_O_5_ [M + Na]^+^: 497.2740; found 497.2764.

N-(4-fluorophenyl)-2-(2-(2-(((2S,2′R,3a’R,5R)-2-isopropyl-5,5′-dimethyl-4′-oxotetrahydro-2′H-spiro[cyclohexane-1,6′-imidazo[1,5-b]isoxazol]-2′-yl)methyl)-6-methylphenoxy)acetyl)hydrazine-1-carboxamide **5a**

Compound **5a** was obtained according to procedure **D**: 4-fluorophenyl isocyanate (40.9 mg, 1.5 eq.), hydrazide **4** (88 mg, 1 eq.). The expected compound was obtained in the form of oil (79%). 1H NMR (CDCl_3_, 400 MHz) δppm: 0.65 (d, 3H, *J* = 6.4 Hz, CH_3_); 0.80 (2d, 6H, *J* = 6.8 Hz, 2CH_3_); 0.84–0.87 (m, 1H); 1.09 (t, 1H, *J* = 12.4 Hz); 1.25–1.35 (m, 2H); 1.55–1.58 (m, 2H); 1.63–1.75 (m, 2H); 1.87–1.99 (m, 1H); 2.29 (s, 3H); 2.50 (dd, 1H, *J* = 8.8 Hz, 11.6 Hz); 2.67 (s, 3H, NCH_3_); 2.72 (dd, 1H, *J* = 3 Hz, 12Hz); 2.88–2.95 (m, 2H); 3.81–3.85 (m, 1H); 3.97 (d, 1H, *J* = 8.4 Hz); 4.29 (d, 1H, *J* = 15.2 Hz); 4.68 (d, 1H, *J* = 15.2 Hz); 6.85 (t, 2H, *J* = 8.4 Hz); 6.98–7.08 (m, 3H); 7.18–7.21 (m, 2H); 7.17 (s, 1H, NH); 7.85 (s, 1H, NH); 9.86 (s, 1H, NH). 13C NMR (CDCl_3_, 100 MHz) δppm: 16.4; 18.1; 21.9; 22.3; 24.1; 24.3; 26.0; 29.7; 29.8; 33.5; 34.5; 38.9; 40.4; 47.9; 66.4; 70.9; 79.1; 90.0; 115.2; 115.4; 121.2; 121.3; 125.1; 128.9; 130.2; 131.0; 131.2; 134.0; 154.6; 154.9; 170.0; 172.7. Anal. calc. for C_32_H_42_FN_5_O_5_ (595.72) (%): C, 64.52; H, 7.11; O, 13.43. Found: C,64.11; H, 6.98; O, 13.31. HRMS, calcd C_32_H_42_FN_5_O_5_ [M + Na]^+^: 618.3068; found 618.3091.

N-(3-trifluoromethyl-4-chlorophenyl)-2-(2-(2-(((2S,2′R,3a’R,5R)-2-isopropyl-5,5′-dimethyl-4′-oxotetrahydro-2′H-spiro[cyclohexane-1,6′-imidazo[1,5-b]isoxazol]-2′-yl)methyl)-6-methylphenoxy)acetyl)hydrazine-1-carboxamide **5b**

Compound **5b** was obtained according to procedure **D**: 4-chloro-3-trifluoromethylphenyl isocyanate (75 mg, 1.5 eq.), hydrazide **4** (100 mg, 1 eq.). The expected compound was obtained in the form of oil (81%).

1H NMR (CDCl_3_, 400 MHz) δppm: 0.65 (d, 3H, *J* = 6.4 Hz, CH_3_); 0.77 (d, 3H, *J* = 6.8 Hz, CH_3_); 0.80 (d, 3H, *J* = 7.2 Hz, CH_3_); 0.81–0.87 (m, 1H); 1.09 (t, 1H, *J* = 12.4 Hz); 1.29–1.34 (m, 2H); 1.54–1.62 (m, 3H); 1.77 (d, 1H, *J* = 12Hz); 1.79–1.89 (m, 1H); 2.31 (s, 3H, CH_3_); 2.50 (dq, 1H, *J* = 9.2 Hz, 11.6 Hz); 2.67 (s, 3H, NCH_3_); 2.73 (dd, 1H, *J* = 2.7 Hz, 12Hz); 2.94–2.96 (m, 2H); 3.81–3.86 (m, 1H); 3.96 (d, 1H, *J* = 8.4 Hz); 4.32 (d, 1H, *J* = 15.2 Hz); 4.75 (d, 1H, *J* = 15.2 Hz); 7.00–7.10 (m, 3H); 7.20 (d, 1H, *J* = 8.4 Hz); 7.34 (dd, 1H, *J* = 2.0 Hz, 8.8 Hz); 7.55 (d, 1H, *J* = 2.0 Hz); 7.96 (s, 1H, NH); 8.06 (s, 1H, NH); 9.92 (s, 1H, NH). 13C NMR (CDCl_3_, 100 MHz) δppm: 16.3; 18.0; 21.8; 22.3; 24.0; 24.3; 26.0; 29.8; 33.7; 34.5; 38.8; 40.4; 47.9; 66.4; 70.9; 77.2; 79.3; 90.1; 117.7; 117.8; 122.7; 123.9; 125.3; 129.1; 130.3; 131.0; 131.2; 131.5; 137.1; 154.2; 154.6; 170.9; 172.7. Anal. calc. for C_33_H_41_ClF_3_N_5_O_5_ (680.17) (%): C, 58.27; H, 6.08; O, 11.76. Found: C, 57.93; H, 5.96; O, 11.68. HRMS, calcd C_33_H_41_ClF_3_N_5_O_5_ [M + Na]^+^: 652.2646; found 652.2671.

N-(3-chlorophenyl)-2-(2-(2-(((2S,2′R,3a’R,5R)-2-isopropyl-5,5′-dimethyl-4′-oxotetrahydro-2′H-spiro[cyclohexane-1,6′-imidazo[1,5-b]isoxazol]-2′-yl)methyl)-6-methylphenoxy)acetyl)hydrazine-1-carboxamide **5c**

Compound **5c** was obtained according to procedure **D**: 3-chlorophenyl isocyanate (49 mg, 1.5 eq.), hydrazide **4** (95 mg, 1 eq.). The expected compound was obtained in the form of oil (85%). 1H NMR (CDCl_3_, 400 MHz) δppm: 0.65 (d, 3H, *J* = 6.8 Hz, CH_3_); 0.78 (d, 3H, *J* = 6.8 Hz, CH_3_); 0.79 (d, 3H, *J* = 6.8 Hz, CH_3_); 0.83–0.87 (m, 1H); 1.08 (t, 1H, *J* = 12.4 Hz); 1.25–1.33 (m, 2H); 1.56–1.58 (m, 2H); 1.62–1.77 (m, 2H); 1.92 (brs, 1H); 2.27 (s, 3H, CH_3_); 2.48 (dd, 1H, *J* = 8.8 Hz, 11.6 Hz); 2.66 (s, 3H, NCH_3_); 2.72 (dd, 1H, *J* = 3 Hz, 12.4 Hz); 2.87–2.95 (m, 2H); 3.81–3.85 (m, 1H); 3.96 (d, 1H, *J* = 8.0 Hz); 4.30 (d, 1H, *J* = 15.2 Hz); 4.64 (d, 1H, *J* = 15.2 Hz); 6.86–6.89 (m, 1H); 6.97–7.04 (m, 5H); 7.38 (s, 1H, NH); 8.11 (s, 1H); 8.13 (s, 1H, NH); 9.96 (s, 1H, NH). 13C NMR (CDCl_3_, 100 MHz) δppm 16.4; 18.1; 21.9; 22.3; 24.0; 24.3; 26.0; 26.9; 29.7; 33.4; 34.4; 38.8; 40.3; 47.8; 66.4; 70.8; 79.0; 90.0; 116.8; 118.9; 122.8; 125.1; 128.9; 129.6; 130.1; 130.9; 131.2; 134.2; 139.4; 154.5; 170.1; 172.8. Anal. calc. for C_32_H_42_ClN_5_O_5_ (612.17) (%): C, 62.79; H, 6.92; O, 13.07. Found: C, 62.29; H, 6.83; O, 12.97. HRMS, calcd C_32_H_42_ClN_5_O_5_ [M + Na]^+^: 634.2772; found 634.2791.

2-(2-(2-(((2S,2′R,3a’R,5R)-2-isopropyl-5,5′-dimethyl-4′-oxotetrahydro-2′H-spiro[cyclohexane-1,6′-imidazo[1,5-b]isoxazol]-2′-yl)methyl)-6-methylphenoxy)acetyl)-N-(naphthalen-1-yl)hydrazine-1-carboxamide **5d**

Compound **5d** was obtained according to procedure **D**: naphthyl isocyanate (64.12 mg, 1.5 eq.), hydrazide **4** (112 mg, 1 eq.). The expected compound was obtained in the form of oil (81%). 1H NMR (CDCl_3_, 400 MHz) δppm 0.63 (d, 3H, *J* = 6.4 Hz, CH_3_); 0.80 (d, 3H, *J* = 6.4 Hz, CH_3_); 0.81 (d, 3H, *J* = 6.4 Hz, CH_3_); 0.84–0.86 (m, 1H); 1.08 (t, 1H, *J* = 12.4 Hz); 1.29–1.36 (m, 3H); 1.64–1.71 (m, 3H); 1.87–1.94 (m, 1H); 2.27 (s, 3H, CH_3_); 2.38 (ddd, 1H, *J* = 3.2 Hz, 8.8 Hz and 12 Hz); 2.66 (s, 3H, NCH_3_); 2.70 (dd, 1H, *J* = 3 Hz, 12Hz); 2.89 (d, 2H, *J* = 6.0 Hz); 3.84 (m, 1H); 3.97 (d, 1H, *J* = 8.4 Hz); 4.29 (d, 1H, *J* = 15.2 Hz); 4.67 (d, 1H, *J* = 15.2 Hz); 6.97–7.07 (m, 3H); 7.41–7.48 (m, 3H); 7.53 (s, 1H, NH); 7.59 (s, 1H, NH); 7.69 (d, 1H, *J* = 8.0 Hz); 7.81 (d, 1H, *J* = 7.6 Hz); 7.85 (dd, 1H, *J* = 4.0 Hz, 9.2 Hz); 8.00 (dd, 1H, *J* = 4.0 Hz, 9.2 Hz); 9.70 (s, 1H, NH). 13C NMR (CDCl_3_, 100 MHz) δppm 16.4; 18.2; 21.9; 22.3; 24.1; 24.3; 26.0; 29.7; 33.4; 34.4; 38.9; 40.4; 47.9; 66.4; 71.0; 78.9; 90.0; 121.2; 121.3; 125.1; 125,8; 125.9; 126.2; 126.4; 128.0; 128.6; 128.9; 130.2; 130.9; 131.2; 132.4; 134.3; 154.6; 155.7; 168.6; 172.8. Anal. calc. for C_36_H_45_N_5_O_5_ (627.79) (%): C, 68.88; H, 7.23; O, 12.74. Found: C, 68.39; H, 7.15; O, 12.61. HRMS, calcd C_36_H_45_N_5_O_5_ [M + Na]^+^: 650.3319; found 650.3339.

N-(4-chlorophenyl)-2-(2-(2-(((2S,2′R,3a’R,5R)-2-isopropyl-5,5′-dimethyl-4′-oxotetrahydro-2′H-spiro[cyclohexane-1,6′-imidazo[1,5-b]isoxazol]-2′-yl)methyl)-6-methylphenoxy)acetyl)hydrazine-1-carboxamide **5e**

Compound **5e** was obtained according to procedure **D**: 4-chlorophenyl isocyanate (55.13 mg, 1.5 eq.), hydrazide **4** (106 mg, 1 eq.). The expected compound is obtained in the form of oil (90%). 1H NMR (CDCl_3_, 400 MHz) δppm: 0.65 (d, 3H, *J* = 6.4 Hz, CH_3_); 0.79 (d, 3H, *J* = 6.4 Hz, CH_3_); 0.78 (d, 3H, *J* = 6.4 Hz, CH_3_); 0.80–0.87 (m, 1H); 1.09 (t, 1H, *J* = 12.4 Hz); 1.29–1.34 (m, 3H); 1.55–1.58 (m, 2H); 1.62–1.69 (m, 2H); 1.75–1.78 (m, 1H); 1.87–1.98 (m, 1H); 2.29 (s, 3H, CH_3_); 2.48 (dq, 1H, *J* = 8.8 Hz, 11.6 Hz); 2.66 (s, 3H, NCH_3_); 2.71 (dd, 1H, *J* = 4 Hz, 12Hz); 2.88–2.95 (m, 2H); 3.81–3.84 (m, 1H); 3.96 (d, 1H, *J* = 8.4 Hz); 4.29 (d, 1H, *J* = 15.6 Hz); 4.68 (d, 1H, *J* = 15.6 Hz); 7.00–7.10 (m, 3H); 7.11 (d, 2H, *J* = 8.8 Hz); 7.82 (s, 1H, NH); 7.87 (s, 1H, NH); 9.85 (s, 1H, NH). 13C NMR (CDCl_3_, 100 MHz) δppm: 16.4; 18.1; 21.0; 21.9; 22.3; 24.1; 24.3; 26.0; 29.8; 30.9; 33.6; 34.5; 38.9; 40.4; 47.9; 66.4; 70.9; 79.1; 90.1; 120.4; 125.1; 128.0; 128.7; 129.0; 130.2; 131.0; 131.2; 136.8; 154.60; 154.62; 170.0; 172.8. Anal. calc. for C_32_H_42_ClN_5_O_5_ (612.17) (%): C, 62.79; H, 6.92; O, 13.07 .Found: C, 62.41; H, 6.69; O, 12.97. HRMS, calcd C_32_H_42_ClN_5_O_5_ [M + Na]^+^: 634.2772; found 634.2789. 

N-(4-bromophenyl)-2-(2-(4-(((2S,2′R, 3a’R, 5R)-2-isopropyl-5,5′-dimethyl-4′-oxotetrahydro-2′H-spiro[cyclohexane-1,6′-imidazo[1,5-b]isoxazol]-2′-yl)methyl)-2-methoxyphenoxy)acetyl)hydrazine-1-carboxamide **5f**

Compound **5f** was obtained according to procedure **D**: 4-bromophenyl isocyanate (62.56 mg, 1.5 eq.), hydrazide **8** (100 mg, 1 eq.). The expected compound was obtained i the form of oil (85%). 1H NMR (CDCl_3_, 400 MHz) δppm: 0.63 (d, 3H, *J* = 6.4 Hz, CH_3_); 0.77 (d, 3H, *J* = 6.0 Hz, CH_3_); 0.79 (d, 3H, *J* = 6.4 Hz); 0.78 (m, 1H); 1.06 (t, 1H, *J* = 12.4 Hz); 1.23–1.26 (m, 2H); 1.30–1.35 (m, 1H); 1.53–1.65 (m, 3H); 1.70–1.73 (m, 1H); 1.81–1.87 (m, 1H); 2.15 (dq, 1H, *J* = 3.2 Hz, 10.8 Hz); 2.59–2.65 (m, 1H); 2.63 (s, 3H, NCH_3_); 2.74 (brd, 2H, *J* = 5.6Hz); 3.73–3.77 (m, 1H); 3.79 (s, 3H, OCH_3_); 3.94 (d, 1H, *J* = 8.4 Hz); 4.58 (s, 2H); 6.68–6.71 (m, 2H); 6.79 (d, 1H, *J* = 8.0 Hz); 7.07 (d, 2H, *J* = 8.8 Hz); 7.18 (d, 2H, *J* = 8.4 Hz); 7.73 (s, 1H, NH); 7.88 (s, 1H, NH); 9.19 (s, 1H, NH). 13C NMR (CDCl_3_, 100 MHz) δppm: 18.3; 21.0; 21.1; 22.1; 22.2; 24.1; 24.3; 26.1; 29.7; 34.4; 38.6; 38.8; 40.3; 47.9; 55.8; 66.3; 69.9; 78.3; 90.0; 113.2; 115.7; 116.7; 120.7; 121.3; 131.6; 134.5; 137.1; 145.6; 149.6; 154.4; 169.1; 171.2. Anal. calc. for C_32_H_42_BrN5O_6_ (672.62) (%): C, 57.14; H, 6.29; O, 14.27. Found: C, 56.89; H, 6.21; O, 14.09. HRMS, calcd C_32_H_42_BrN5O_6_ [M + Na]^+^: 694.2216; found 694.2229.

N-(4-fluorophenyl)-2-(2-(4-(((2S,2′R,3a’R,5R)-2-isopropyl-5,5′-dimethyl-4′-oxotetrahydro-2′H-spiro [cyclohexane-1,6′-imidazo[1,5-b]isoxazol]-2′-yl)methyl)-2-methoxyphenoxy)acetyl)hydrazine-1-carboxamide **5g**

Compound **5g** was obtained according to procedure **D**: 4-fluorophenyl isocyanate (43.38 mg, 1.5 eq.), hydrazide **8** (100 mg, 1 eq.). The expected compound was obtained in the form of oil (86%). 1H NMR (CDCl_3_, 400 MHz) δppm :0.71 (d, 3H, *J* = 6.4 Hz, CH_3_); 0.84 (d, 3H, *J* = 6.4 Hz, CH_3_); 0.85 (d, 3H, *J* = 6.8 Hz, CH_3_); 0.90 (m, 1H); 1.13 (t, 1H, *J* = 12.4 Hz); 1.25 (t, 1H, *J* = 7.2 Hz); 1.31 (dd, 1H, *J* = 2.8 Hz, 11.6 Hz); 1.35–1.42 (m, 1H); 1.58–1.79 (m, 4H); 1.92–1.94 (m, 1H); 2.21 (q, 1H, *J* = 10.8 Hz); 2.66–2.73 (m, 1H); 2.69 (s, 3H, NCH_3_); 2.80 (d, 2H, *J* = 6.4 Hz); 3.80–3.84 (m, 1H); 3.86 (s, 3H, OCH_3_); 4.00 (d, 1H, *J* = 8.4 Hz); 4.65 (s, 2H); 6.75–6.78 (m, 2H); 6.85–6.89 (m, 2H); 7.20–7.23 (m, 2H); 7.61 (s, 1H, NH); 7.69 (s, 1H, NH); 9.19 (s, 1H, NH). 13C NMR (CDCl_3_, 100 MHz) δppm: 18.4; 22.2; 22.3; 24.1; 24.3; 26.1; 29.7; 34.5; 38.7; 38.8; 40.4; 48.0; 55.9; 66.3; 70.0; 78.3; 90.0; 113.3; 115.3; 115.5; 116.7; 121.3; 121.4; 121.5; 133.7, 133.8; 134.6; 145.7; 149.6; 154.8; 169.0; 172.7. Anal. calc. for C_32_H_42_FN_5_O_6_ (611.72) (%): C, 62.83; H, 6.92; O, 15.69. Found: C, 62.33; H, 6.88; O,15.55. HRMS, calcd C_32_H_42_FN_5_O_6_ [M + Na]^+^: 634.3017 found 634.3024.

### 3.2. α-Amylase and α-Glucosidase Inhibition Assays

The inhibitory potential of the synthesized analogs against human pancreatic α-amylase and human lysosomal acid-α-glucosidase enzymes was carried out using the same protocol as described previously with slight modification [20]. Acarbose was used as standard. The percent of inhibition for both enzymes was calculated via the following formula:

$$\%\;inhibition=\frac{\mathrm{Abs}\left(\mathrm{blank}\right)-\mathrm{Abs}\left(\mathrm{test}\;\mathrm{compoud}\right)}{\mathrm{Abs}\;\left(\mathrm{blank}\right)}\times 100$$

The results were expressed as IC_50_ (μM), and all the experiments were carried out in triplicates.

### 3.3. Kinetic Studies

The kinetic studies were carried out based on the above IC50 values. In vitro assays were performed for the most potent inhibitor, **5d**, to determine its inhibition mode towards both enzymes. A volume of 20 μL was incubated for 15 min at 30 °C using varying concentrations of the inhibitor 5d against α-amylase (0, 5, 25, and 35 μM) and α-glucosidase (0, 30, 60, and 95 mM) with varying concentrations of inhibitor substrate (starch) in the range 0.3 to 8 mM, whereas concentrations for the inhibitor substrate (*p*-nitrophenyl-α-D-glucopyranoside, PNPg) were in the range 0.1–1.3 mM, respectively. This was conducted according to the same protocol as previously described, with slight modifications [57,58]. The type of enzyme inhibition was assessed by preparing Lineweaver–Burk plots of the inverse of the velocities (1/V) versus the inverse of the substrate’s concentration 1/[S] mM^−1^, and the change in absorbance was measured spectrophotometrically at 405 nm. The constant of inhibition, Ki, was determined through the secondary plot of the slope versus the inhibitor concentration.

### 3.4. Molecular Docking Study

The methodology followed in this study for modeling, using the α-glucosidase enzyme (PDB code: 3W37) and the α-amylase enzyme (PDB code: 2QV4), was similar to that described previously [59,60,61,62]. The protein structure was created and optimized using the Schrödinger software (Schrodinger, LLC, New York, USA, 2008) package’s protein production wizard. Crystal-bound water molecules and other molecules were removed. Water molecules and other heteroatoms were eliminated from the crystallographic water molecules. Hydrogens were added as needed, and bond orders were given correspondingly. Formal charges, as well as side and backbone chains, were rectified. Formal charges, as well as side and backbone chains, have been corrected. To reduce steric conflicts in the protein structure, the produced protein structure was minimized using the OPLS3 force field. Following that, the produced protein was examined for grid formation using Glide’s “Receptor Grid Generation” module (Grid-Based Ligand Docking with Energetics). The chirality, ionization states, ring conformations, and tautomers of the input 4c structure were investigated using the LigPrep tool. Standard precision docking methodology was employed with the default Force Field OPLS 2005, and the docking score was used to perform an extensive investigation of ligand binding affinities.

### 3.5. Molecular Dynamic (MD) Simulation

To investigate the stability of the lead compound **5d** when interacting with proteins 3W37 and 2QV4, a molecular dynamics (MD) simulation was conducted, following previously established protocols [63,64,65]. The simulation employed the Desmond MD tool and utilized the OPLS-3e force field. Computational tasks were executed on an HP Z2 G2 Tower workstation running Ubuntu 18.04, equipped with an NVIDIA Quadro 6000 4GB GPU. Initially, the ligand–protein complex, obtained from the Glide software (GLIDE, Schrodinger, LLC, New York, NY, USA, 2008), was imported into Schrodinger’s Maestro interface. The complex was then positioned at the center of an orthorhombic box, ensuring a minimum distance of 10 cm between the box edges. SPC water molecules were added to solvate the system, and counter ions (Na^+^ and Cl^−^) were included to neutralize the charges. To mimic physiological conditions, a salt concentration of 0.15 M NaCl was set using the Desmond System Builder panel. Following system preparation, energy minimization was performed using the OPLS3e force field, with 2000 iterations and a convergence criterion of 1 kcal/mol, to eliminate any electronic conflicts within the protein structures. For the production MD simulation, a duration of 100 ns was chosen, with 1000 steps, employing the NPT ensemble at a temperature of 298 K and 1 bar pressure. Temperature and pressure were maintained using the Nose–Hoover Chain thermostat algorithm and the Martyna–Tobias–Klein barostat algorithm, respectively. Finally, Desmond’s Simulation Interaction Diagram (SID) was utilized to analyze the MD trajectories and predict the binding orientation of the ligand within the protein complexes.

### 3.6. ADMET Property Predictions

The prediction of absorption, distribution, metabolism, elimination, and toxicity (ADMET) properties of the most active(s) compound(s) was carried out online through pkCSM software (2015), https://biosig.lab.uq.edu.au/pkcsm/, accessed on 14 September 2023.

### 3.7. Statistical Analysis

SPSS 19 (SPSS Ltd., Woking, United Kingdom) was used for the statistical analysis of the resulting experimental data. The Tukey test was used for the comparison of averages. The *p*-value < 0.05 was used to show the statistical significance of the results.

## 4. Conclusions

In summary, we have successfully designed, synthesized, and characterized novel enantiopure isoxazolidine derivatives through 1,3-diploar cycloaddition. Their enzymatic inhibition effects revealed that **5d** is the most active compound to target α-amylase (IC_50_ = 53.03 ± 0.106 µM) and α-glucosidase (IC_50_ = 94.33 ± 0.282 µM) enzymes. AMDET, MD, and dynamic simulation properties of **5d** were assessed via computational tools. The binding interactions of **5d** within the active site of both enzymes were confirmed through kinetic (competitive manner) and in silico studies. The MD and dynamic simulation studies strongly indicate that **5d** possesses a unique capability to effectively target both α-amylase and α-glucosidase enzymes simultaneously. This dual-targeting potential holds great promise in the realm of antidiabetic drug development. By offering a multifaceted approach to tackling the disease, **5d** emerges as a compelling candidate for further research and development efforts in the quest for more effective antidiabetic medications.

## Figures and Tables

**Table 1:** Coupling constants between H3-H4 and H4-H5 protons.

Entry	Structure	Coupling Constant	Literature [20]
**5a**	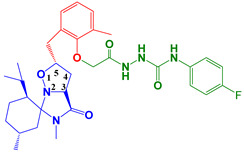	*J*_3,4_ (*cis*) = 8.4 Hz *J*_3,4_ (*trans*)~0 Hz *J*_4,5_ (*trans*) = 8.8 Hz *J*_4,5_ (*cis*) = 3.0 Hz	*J*_3,4_ (*cis*) ≥ 6.6 Hz *J*_3,4_ (*trans*)~0 Hz *J*_4,5_ (*trans*) > 8 Hz *J*_4,5_ (*cis*) < 6 Hz
**5b**	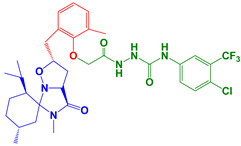	*J*_3,4_ (*cis*) = 8.4 Hz *J*_3,4_ (*trans*)~0 Hz *J*_4,5_ (*trans*) = 9.2 Hz	
**5c**	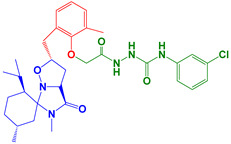	*J*_3,4_ (*cis*) = 8.0 Hz *J*_3,4_ (*trans*)~0 Hz *J*_4,5_ (*trans*) = 8.8 Hz *J*_4,5_ (*cis*) = 3.0 Hz	
**5d**	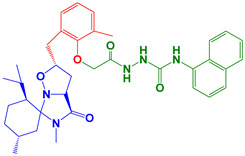	*J*_3,4_ (*cis*) = 8.4 Hz *J*_3,4_ (*trans*)~0 Hz *J*_4,5_ (*trans*) = 8.8 Hz *J*_4,5_ (*cis*) = 3.2 Hz	
**5e**	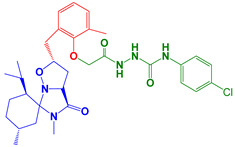	*J*_3,4_ (*cis*) = 8.4 Hz *J*_3,4_ (*trans*)~0 Hz *J*_4,5_ (*trans*) = 8.8 Hz *J*_4,5_ (*cis*) = 4.0 Hz	
**5f**	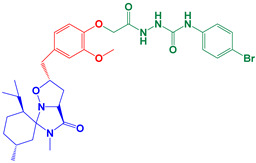	*J*_3,4_ (*cis*) = 8.4 Hz *J*_3,4_ (*trans*)~0 Hz *J*_4,5_ (*cis*) = 3.2 Hz	
**5g**	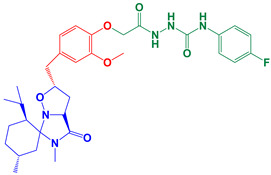	*J*_3,4_ (*cis*) = 8.4 Hz *J*_3,4_ (*trans*)~0 Hz	

**Table 2:** α-Amylase and α-glucosidase inhibitory effects (IC50 in μM) of synthesized compounds. The results are expressed in mean values ± standard error (or standard error); **5a–g**: The means of the same column with a different letter are significantly different (P < 0.05). The means of the same column with the same letter are not significantly different (P > 0.05).

Entry	α-Amylase IC_50_ (μM)	α-Glucosidase IC_50_ (μM)
**5a**	120.3 ± 0.306 ^c^	152.3 ± 0.367 ^c^
**5b**	67.4 ± 0.202 ^a^	118.9 ± 0.325 ^ab^
**5c**	92.28 ± 0.276 ^b^	120.9 ± 0.333 ^b^
**5d**	53.03 ± 0.106 ^a^	94.33 ± 0.282 ^a^
**5e**	232.8 ± 0.517 ^e^	258.7 ± 0.521 ^e^
**5f**	183.8 ± 0.498 ^d^	204.6 ± 0.41 ^d^
**5g**	134.8 ± 0.411 ^c^	170.8 ± 0.358 ^c^
Acarbose	296.6 ± 0.825	780.4 ± 0.346

**Table 3:** ADMET properties of the synthesized compounds using pkCSM.

Entry	5d	Reference
Absorption		
Water solubility	−4.275	-
Caco2 permeability	0.857	>0.9
Intestinal absorption (human)	86.437	<30% is poorly
Skin Permeability (log Kp)	−2.736	>−2.5 is low
Distribution		
VDss (human)	−0.048	Low is <−0.15, High is >0.45
Fraction unbound (human)	0.024	-
BBB permeability	−1.395	Poorly is <−1, High is >0.3
CNS permeability	−2.131	Penetrate is >−2, Unable is <−3
Metabolism		
CYP2D6 substrate	Yes	No
CYP3A4 substrate	Yes	-
CYP1A2 inhibitior	No	No
CYP2C19 inhibitior	Yes	No
CYP2C9 inhibitior	Yes	No
CYP2D6 inhibitior	No	No
CYP3A4 inhibitior	Yes	No
Excretion		
Total clearance	−0.065	-
Renal OCT2 substrate	No	-
Toxicity		
AMES toxicity	No	No
Max. tolerated dose (human)	0.216	Low is ≤0.477, High is >0.477
hERG I inhibitor	No	No
Oral Rat Acute Toxicity (LD50)	3.006	-
Oral Rat Chronic Toxicity (LOAEL)	0.632	-
Skin sensitisation	No	No

**Table 4:** Glide docking score (in Kcal/mol unit) of synthesized derivatives in the active sites of α-amylase enzyme (PDB code: 2QV4) and α-glucosidase enzyme (PDB code: 3W37).

Compounds	α-Amylase Enzyme (PDB Code: 2QV4)	α-Glucosidase Enzyme (PDB Code: 3W37)
**5a**	−5.355	−5.329
**5b**	−5.607	−5.343
**5c**	−5.245	−5.55
**5d**	−5.623	−5.373
**5e**	−3.315	−3.039
**5f**	−4.451	−3.276
**5g**	−5.367	−4.415

## Data Availability

Data are contained within the article and Supplementary Materials.

## Supplementary Materials

The following supporting information can be downloaded at https://www.mdpi.com/article/10.3390/molecules29020305/s1, NMR spectra for compounds **4**, **8**, and **5a–g**.
