# Supplementary material for: In Silico Design, Synthesis, and Evaluation of Novel Enantiopure Isoxazolidines as Promising Dual Inhibitors of α-Amylase and α-Glucosidase
Source: Molecules. 2024 Jan 6;29(2):305. doi: 10.3390/molecules29020305 (PMC10818600; doi:10.3390/molecules29020305)
Supplement: Supplementary file 1 [file molecules-29-00305-s001.zip › molecules-2715611-supplementary.pdf]

Supporting Information

In Silico Design, Synthesis and Evaluation of Novel Enantiopure Isoxazolidines as Dual α-Amylase and α-Glucosidase Promising Inhibitors

Fahad Alhawday,[1] Fahad Alminderej,[1] Siwar Ghannay,[1] Bechir Hammami,[1,2] Abuzar E. A. E. Albadri,[1] Adel Kadri,[3,4,*] Kaiss Aouadi[1,5, *]

[1] Department of Chemistry, College of Science, Qassim University, Buraidah 51452, Saudi Arabia; 431114194@qu.edu.sa (F.H); f.alminderej@qu.edu.sa (F.A.); s.ghannay@qu.edu.sa (S.G.); b.hammami@qu.edu.sa (B.H.); aa.albadri@qu.edu.sa (A.A.); K.AOUADI@qu.edu.sa (K.A.)
[2] Faculty of Sciences of Bizerte FSB, University of Carthage, 7021 Jarzouna, Tunisia.
[3] Faculty of Science of Sfax, Department of Chemistry, University of Sfax, B.P. 1171, 3000 Sfax, Tunisia; lukadel@yahoo.fr (A.K)
[4] Faculty of Science and Arts in Baljurashi, Al-Baha University, P.O. Box (1988). Al-Baha 65527, Saudi Arabia.
[5] Department of Chemistry, Laboratory of Heterocyclic Chemistry Natural Product and Reactivity/CHPNR, Faculty of Science of Monastir, University of Monastir, Avenue of the Environment, Monastir 5019, Tunisia

* Correspondence: Pr. Kaiss Aouadi, email: K.AOUADI@qu.edu.sa; Pr. Adel Kadri, email: lukadel@yahoo.fr

1H, 13C NMR of compound **4**	**2-3**
1H, 13C NMR of compound **8**	**4-5**
1H, 13C NMR of compound **5a**	**6-7**
1H, 13C NMR of compound **5b**	**8-9**
1H, 13C NMR of compound **5c**	**10-11**
1H, 13C NMR of compound **5d**	**12-13**
1H, 13C NMR of compound **5e**	**14-15**
1H, 13C NMR of compound **5f**	**16-17**
1H, 13C NMR of compound **5g**	**18-19**

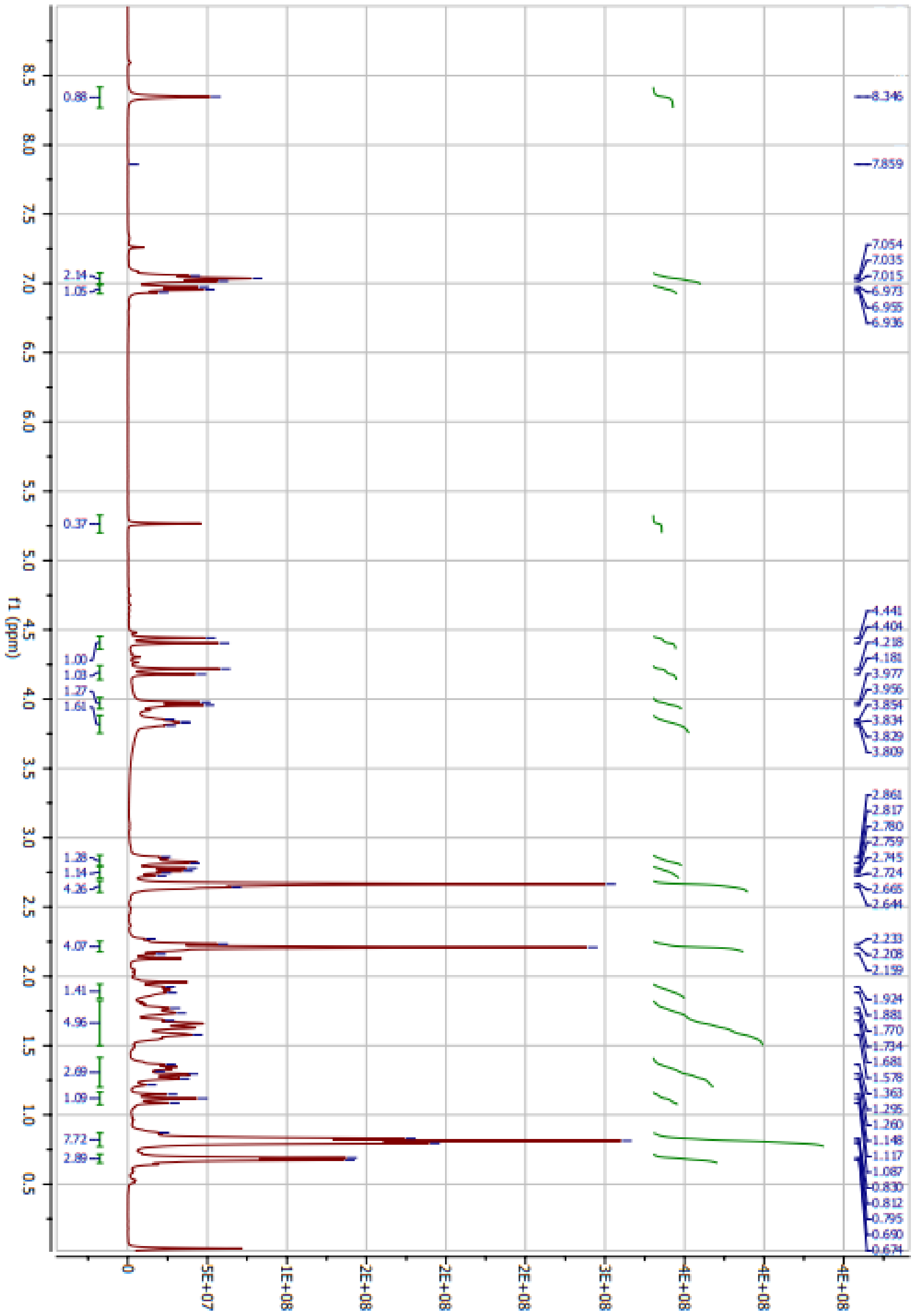

1H NMR for compound **4**

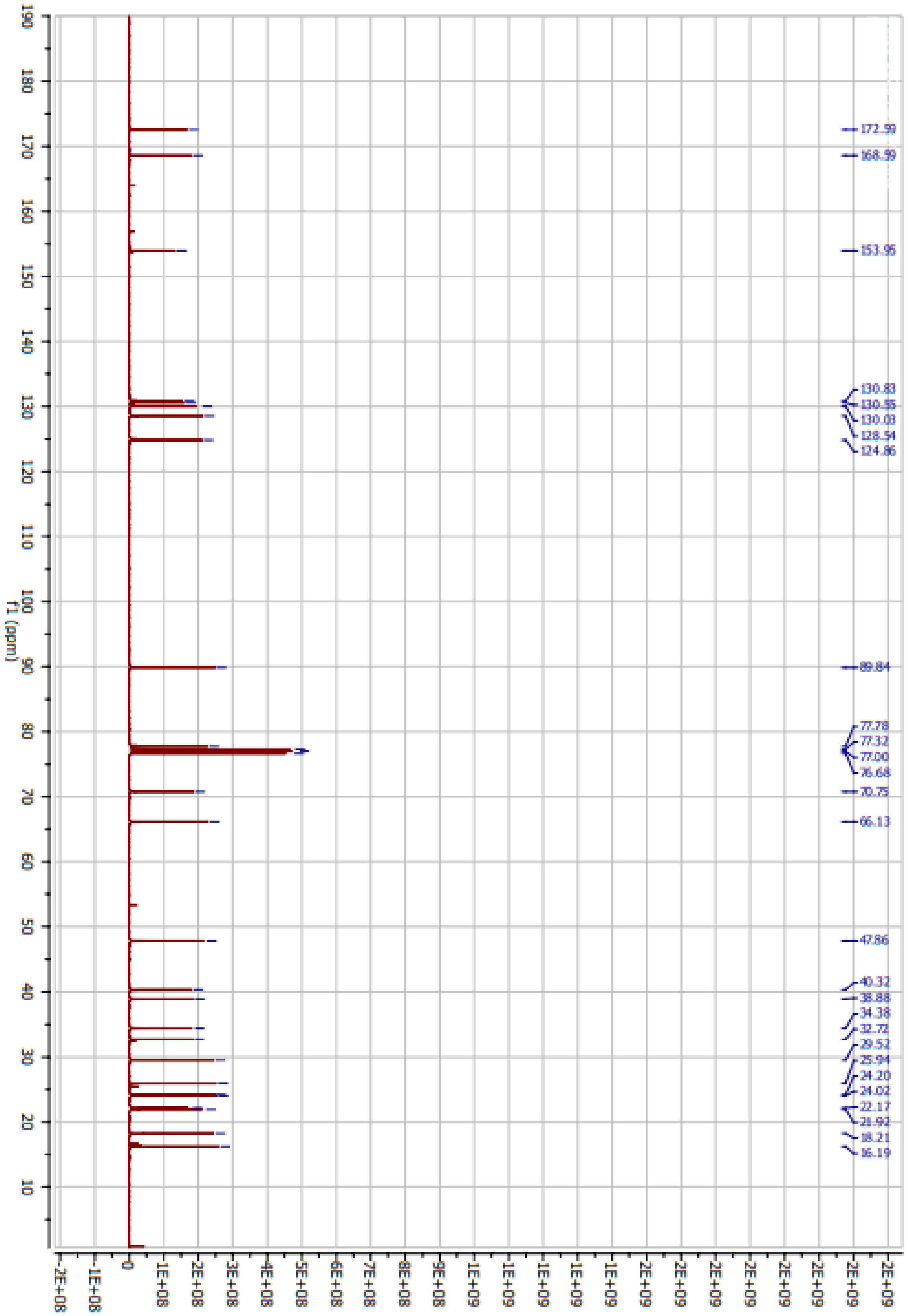

13C NMR for compound **4**

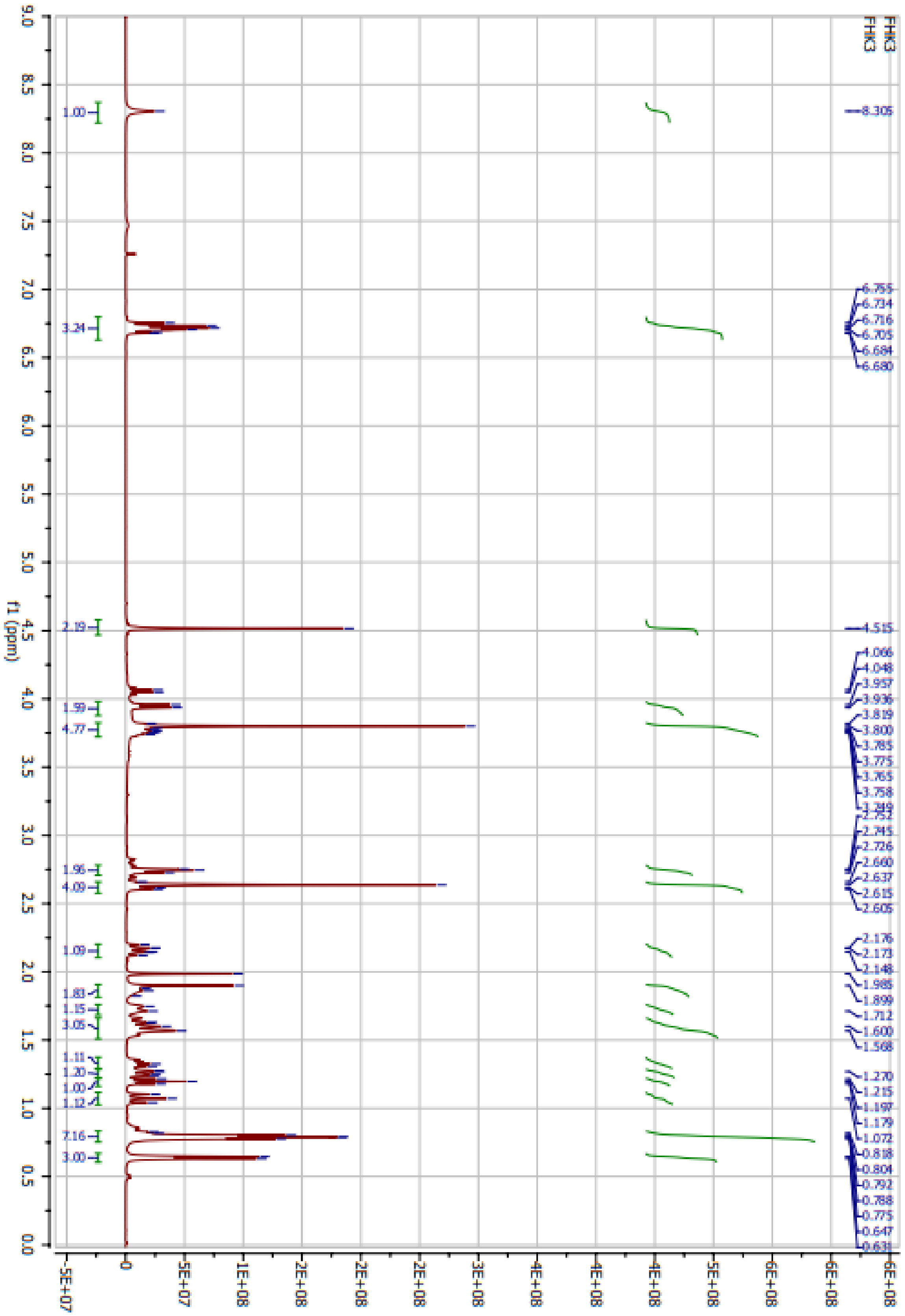

1H NMR for compound **8**

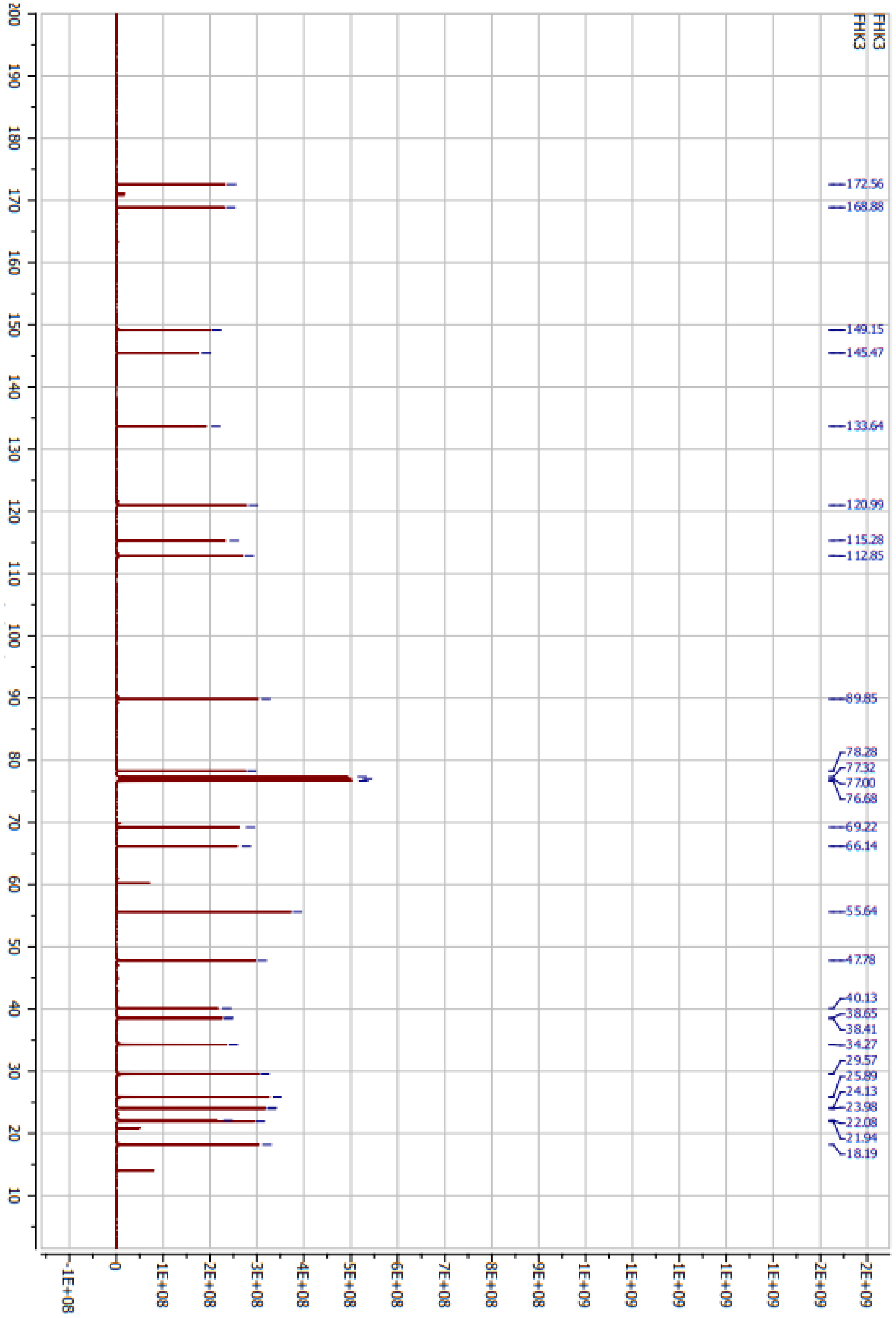

13C NMR for compound **8**

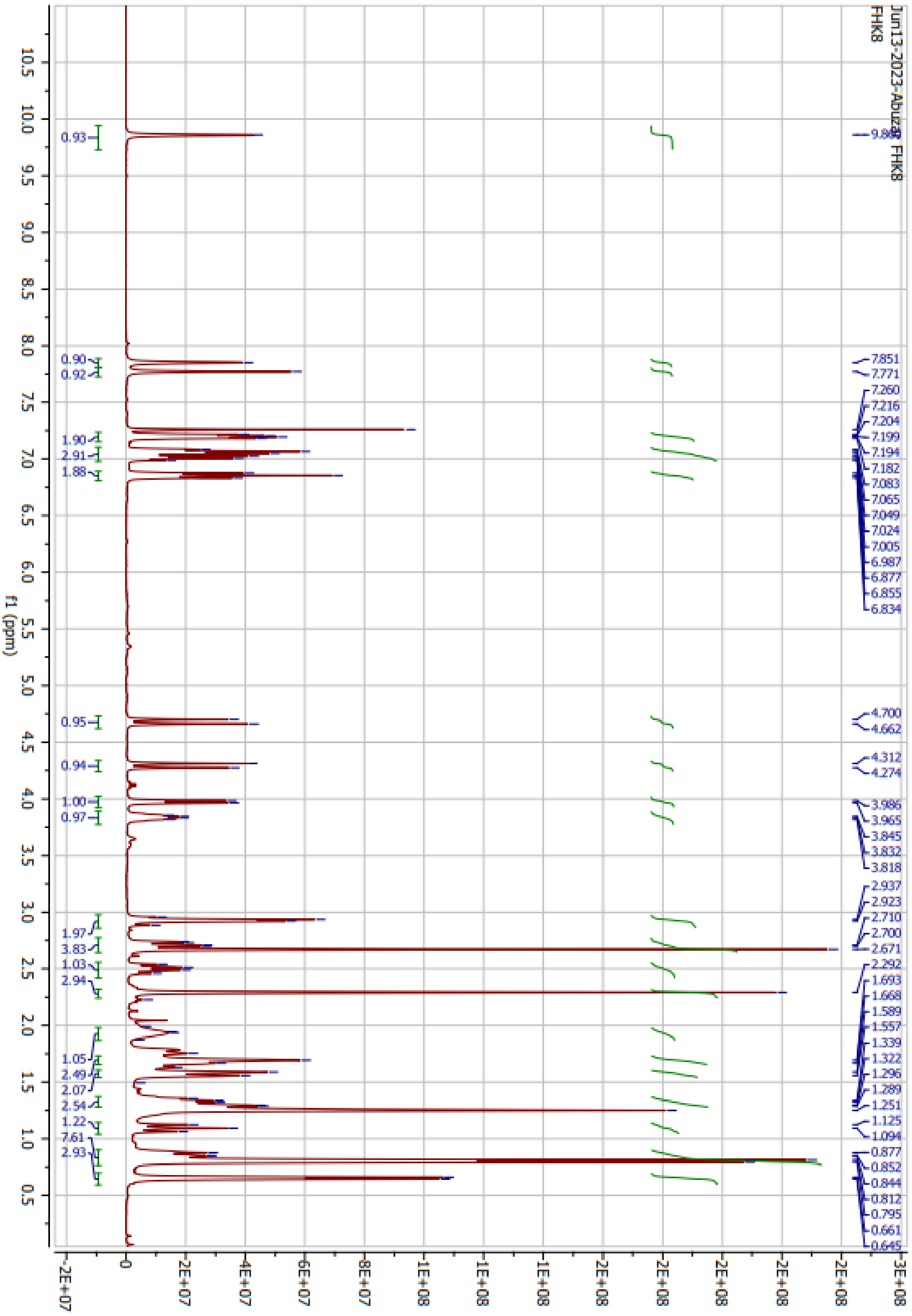

1H NMR for compound **5a**

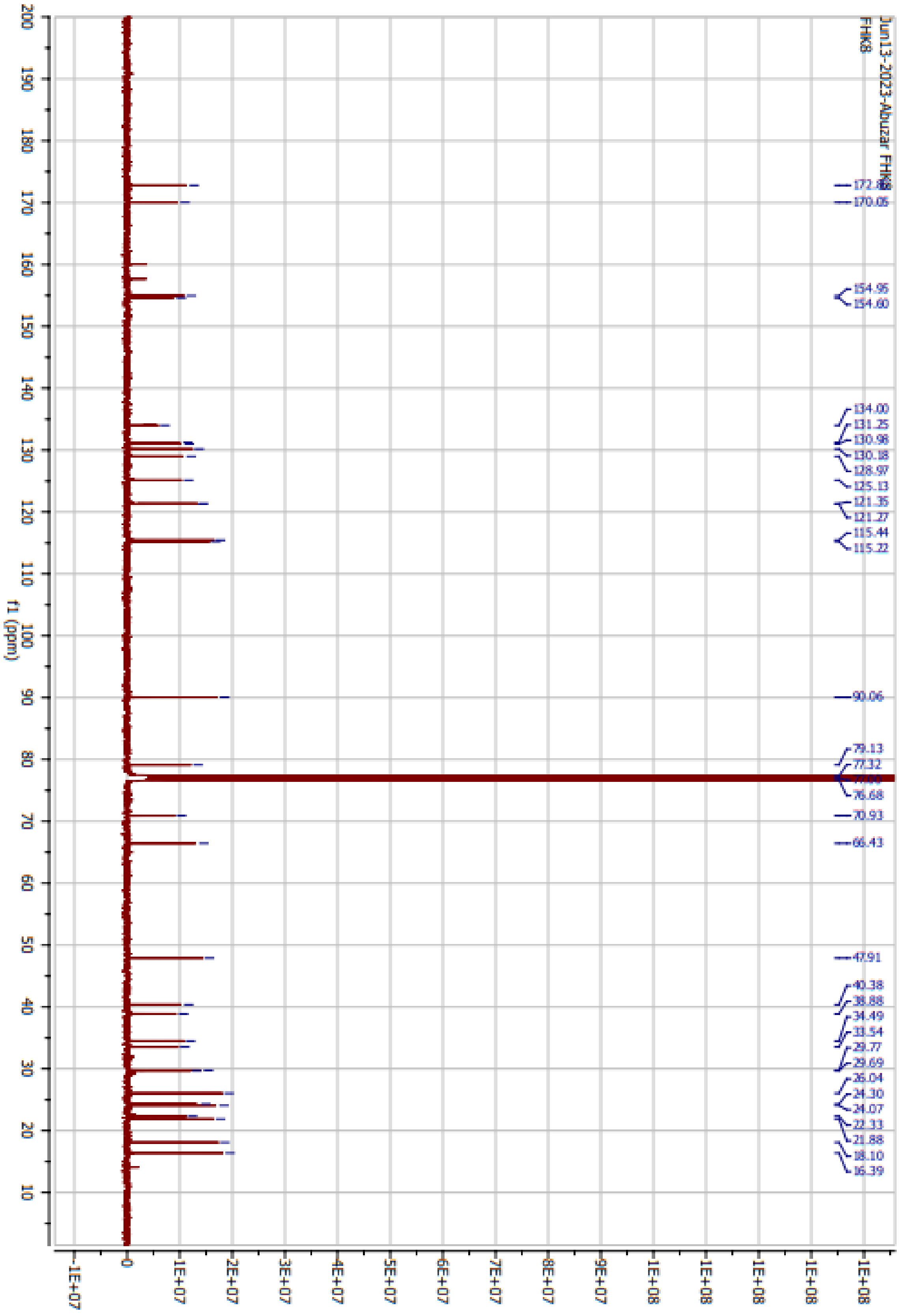

13C NMR for compound **5a**

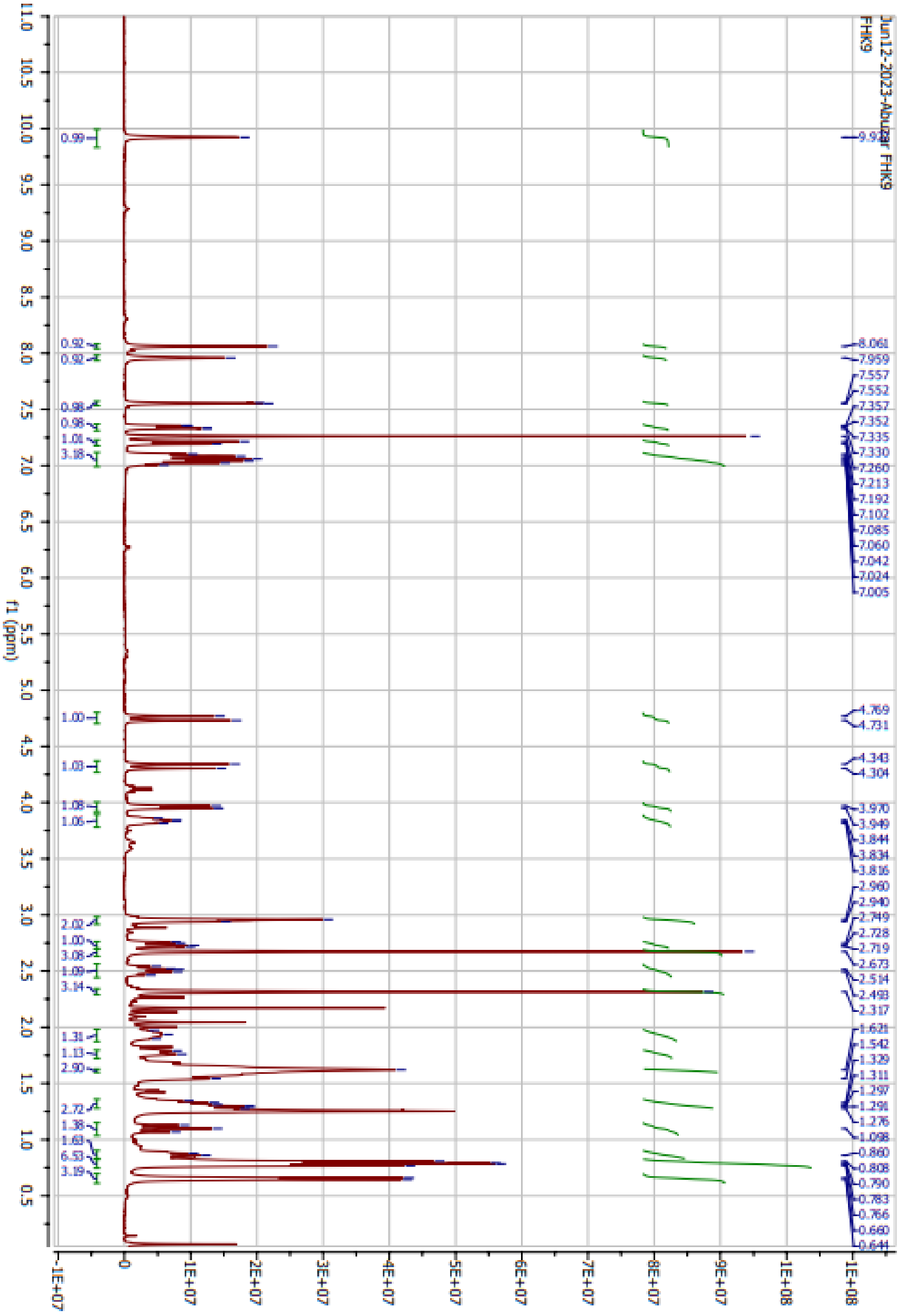

1H NMR for compound **5b**

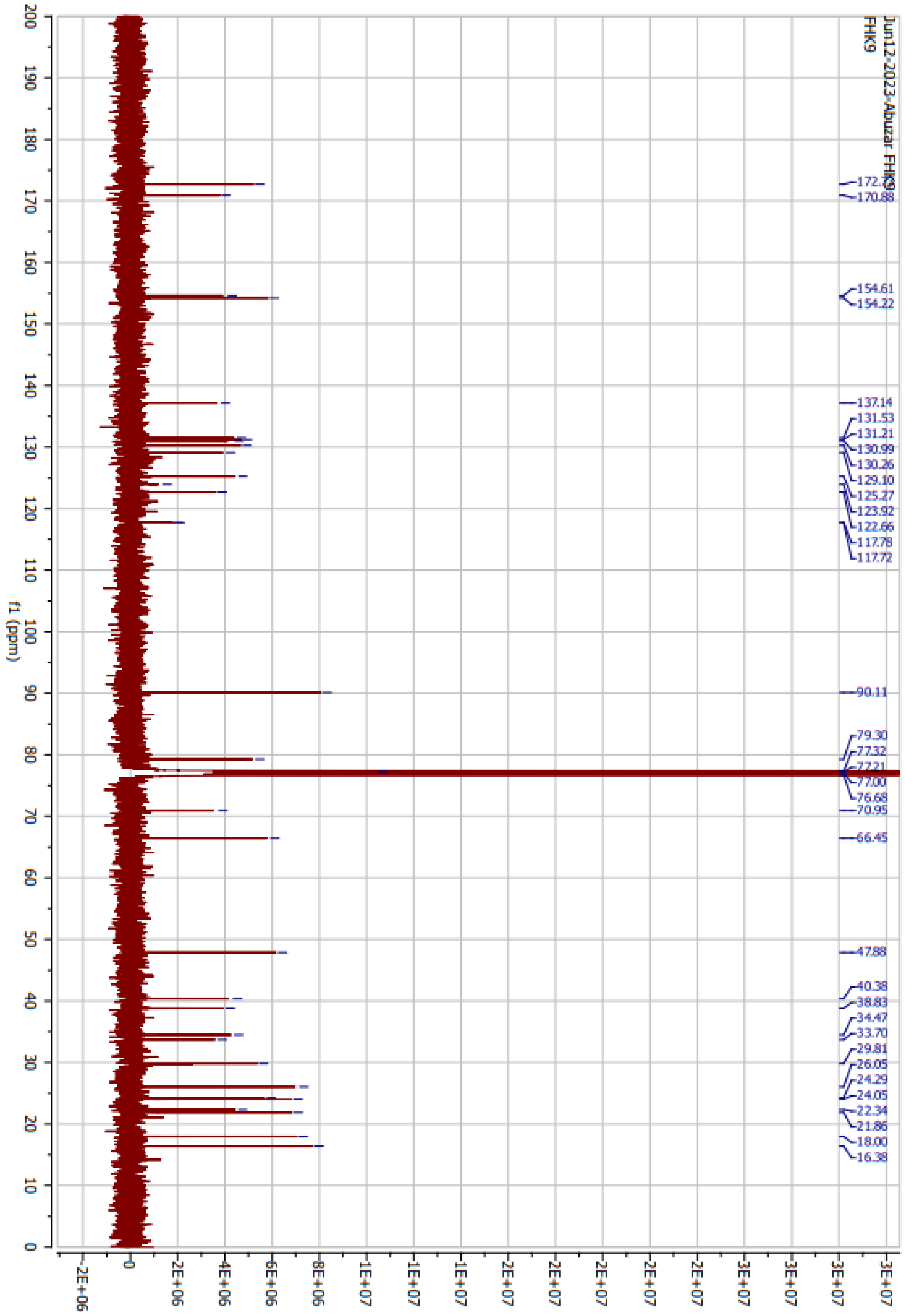

13C NMR for compound **5b**

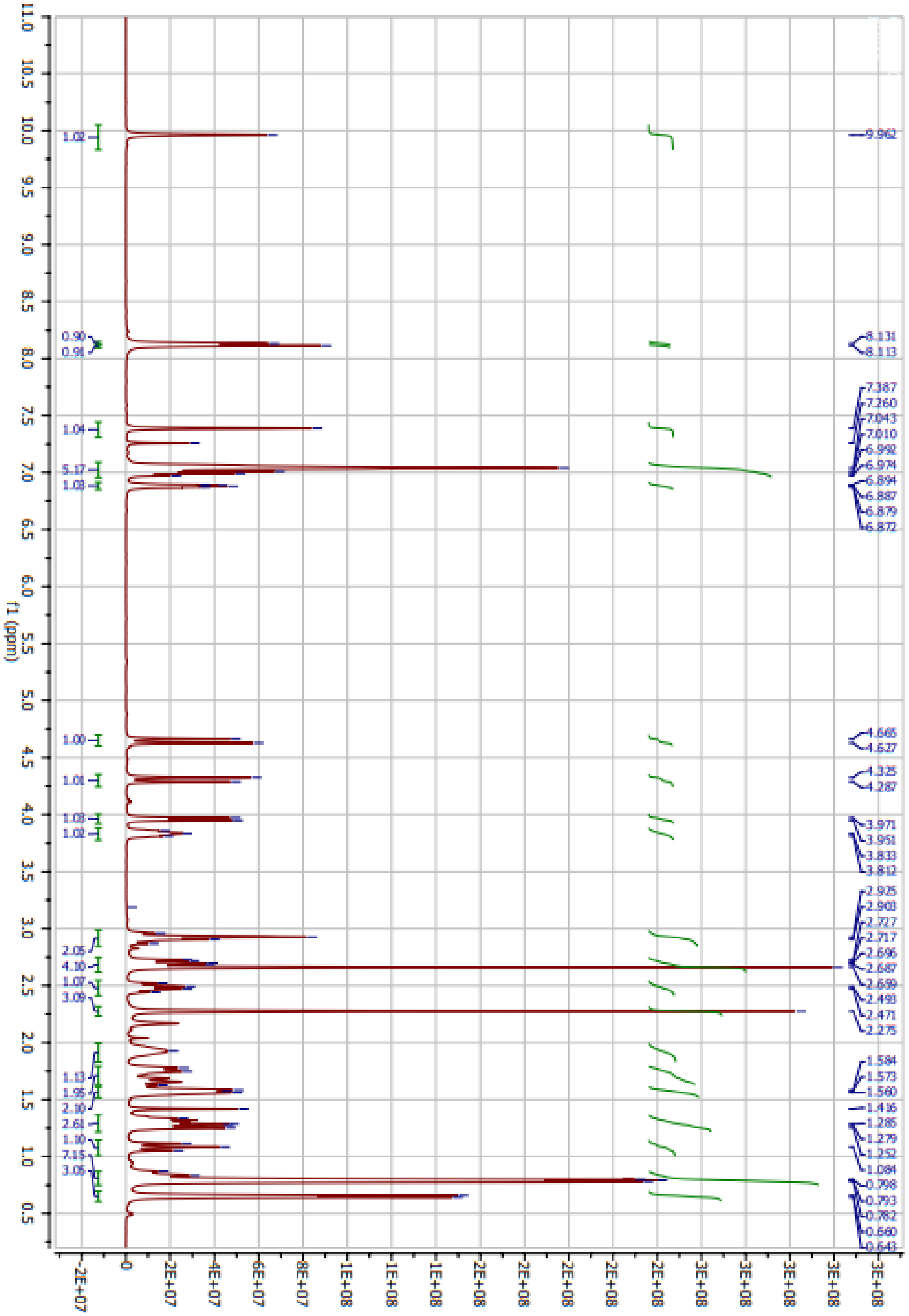
f1 (ppm)

1H NMR for compound **5c**

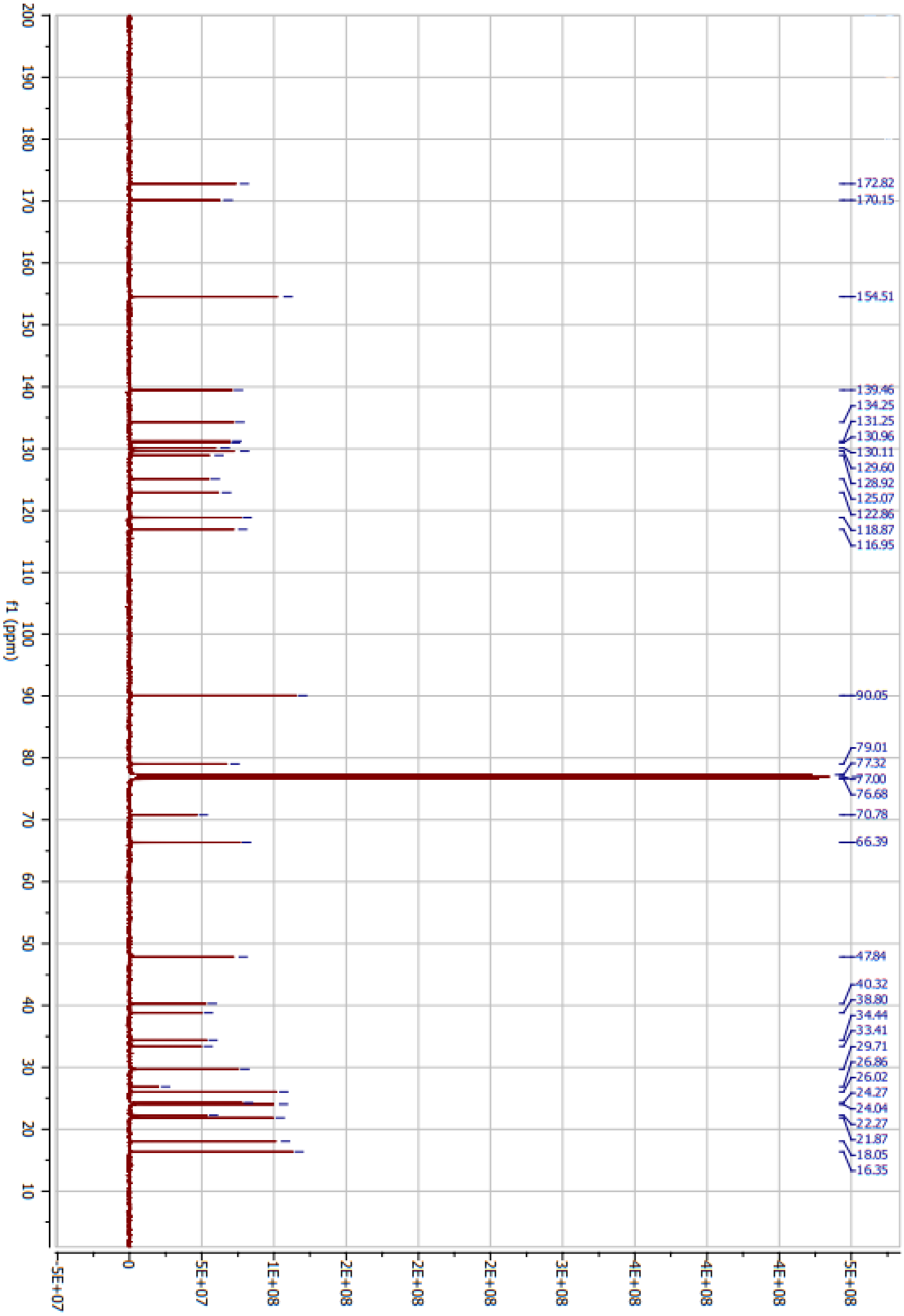

13C NMR for compound **5c**

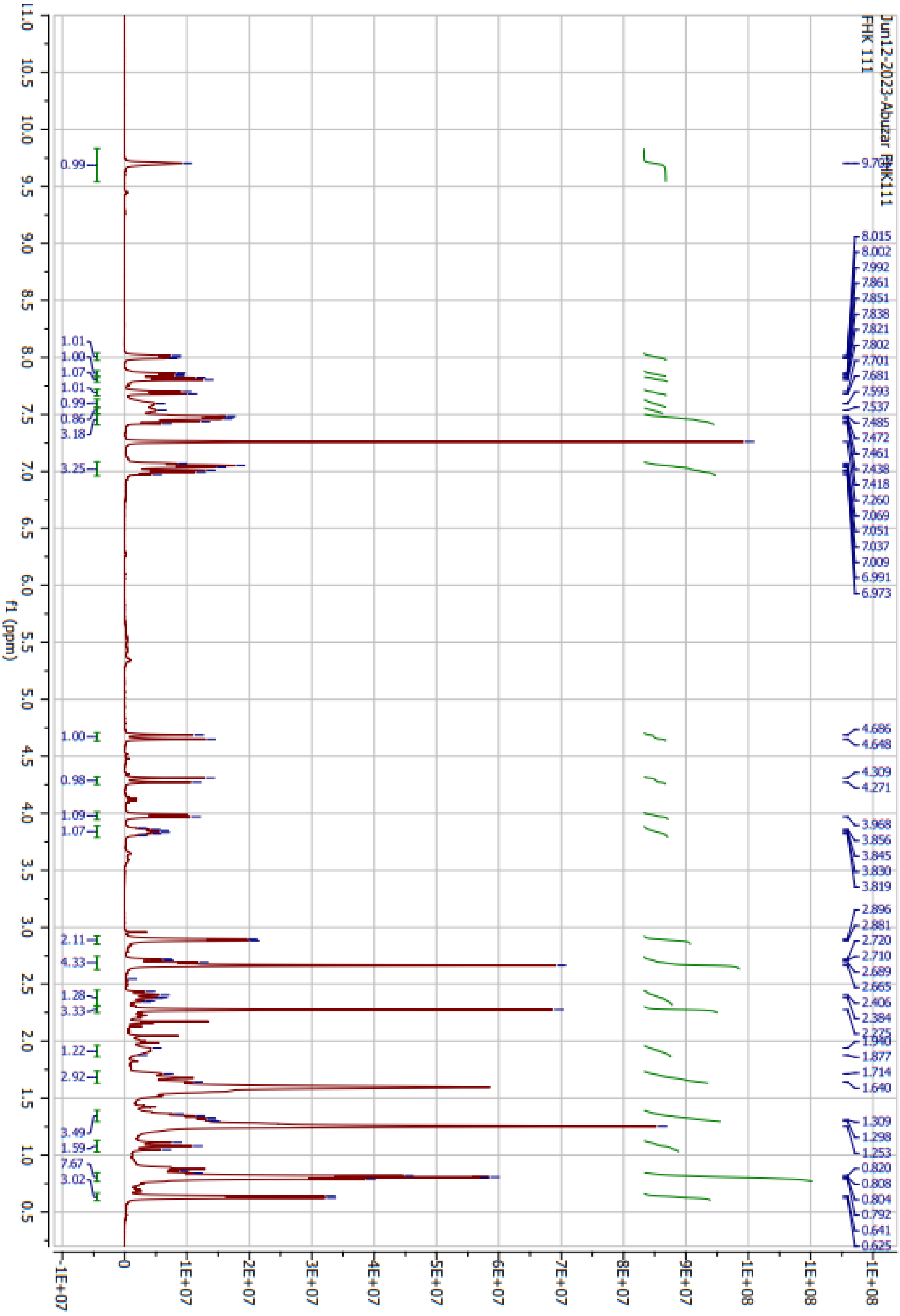

1H NMR for compound **5d**

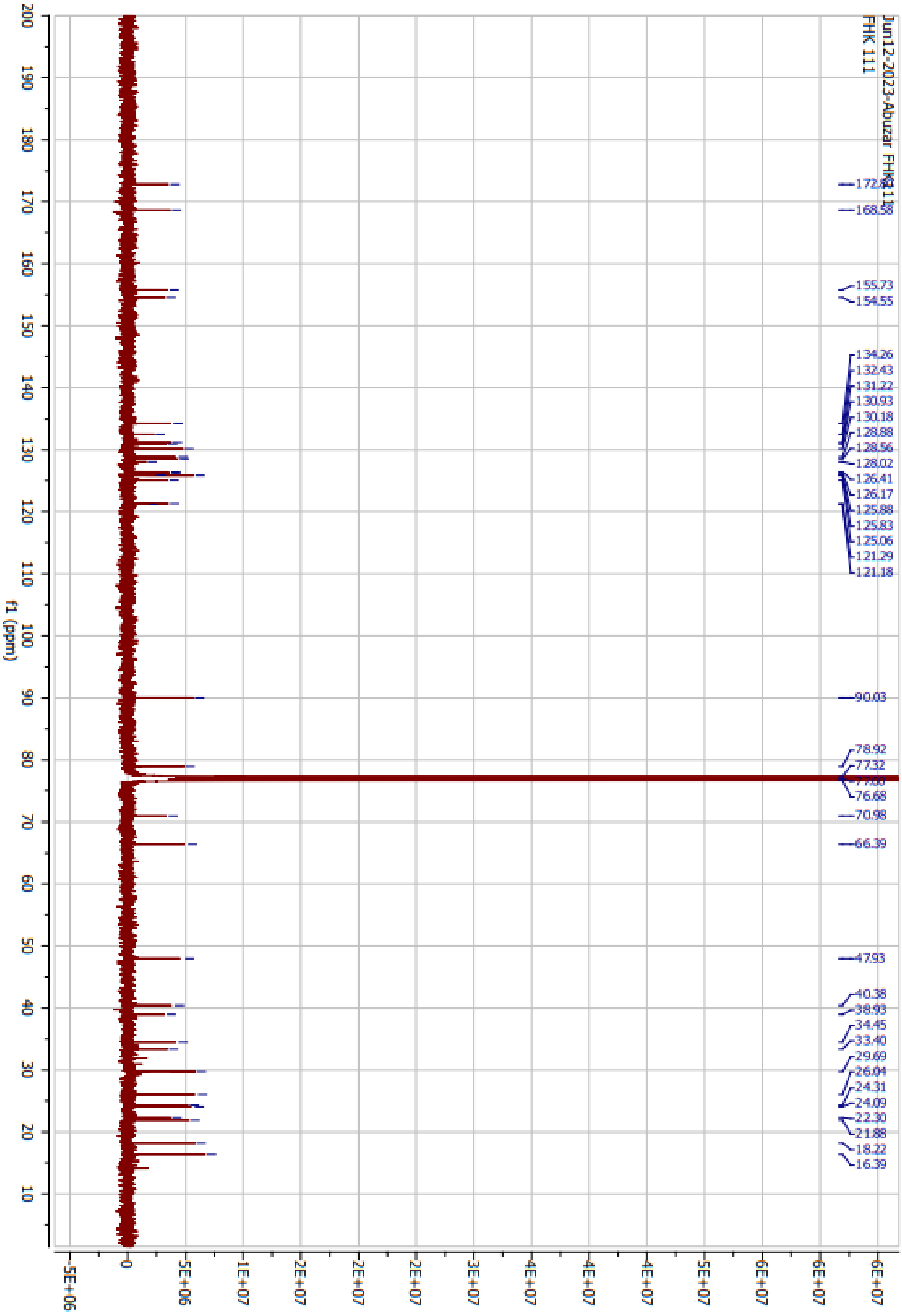

13C NMR for compound **5d**

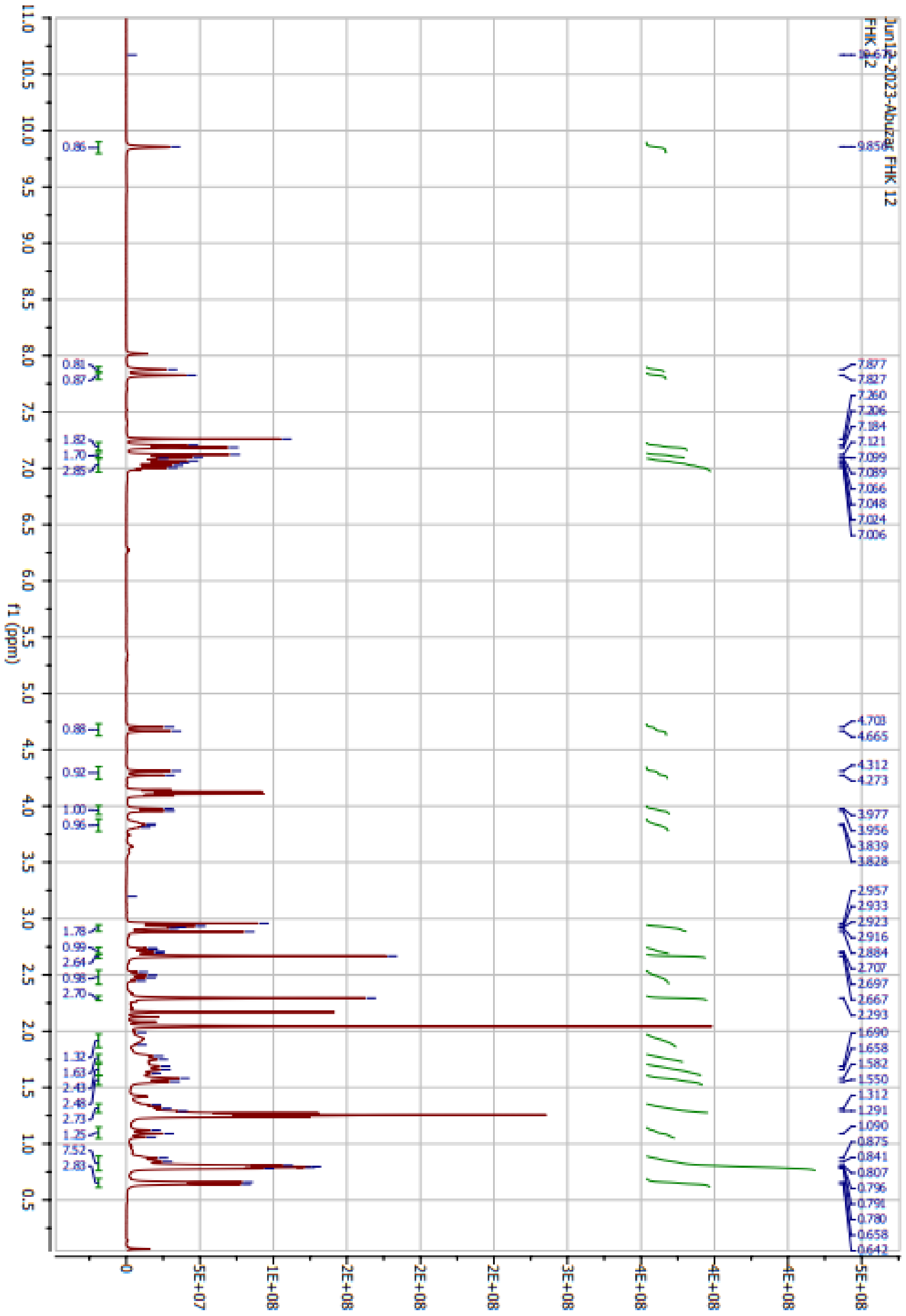

1H NMR for compound **5e**

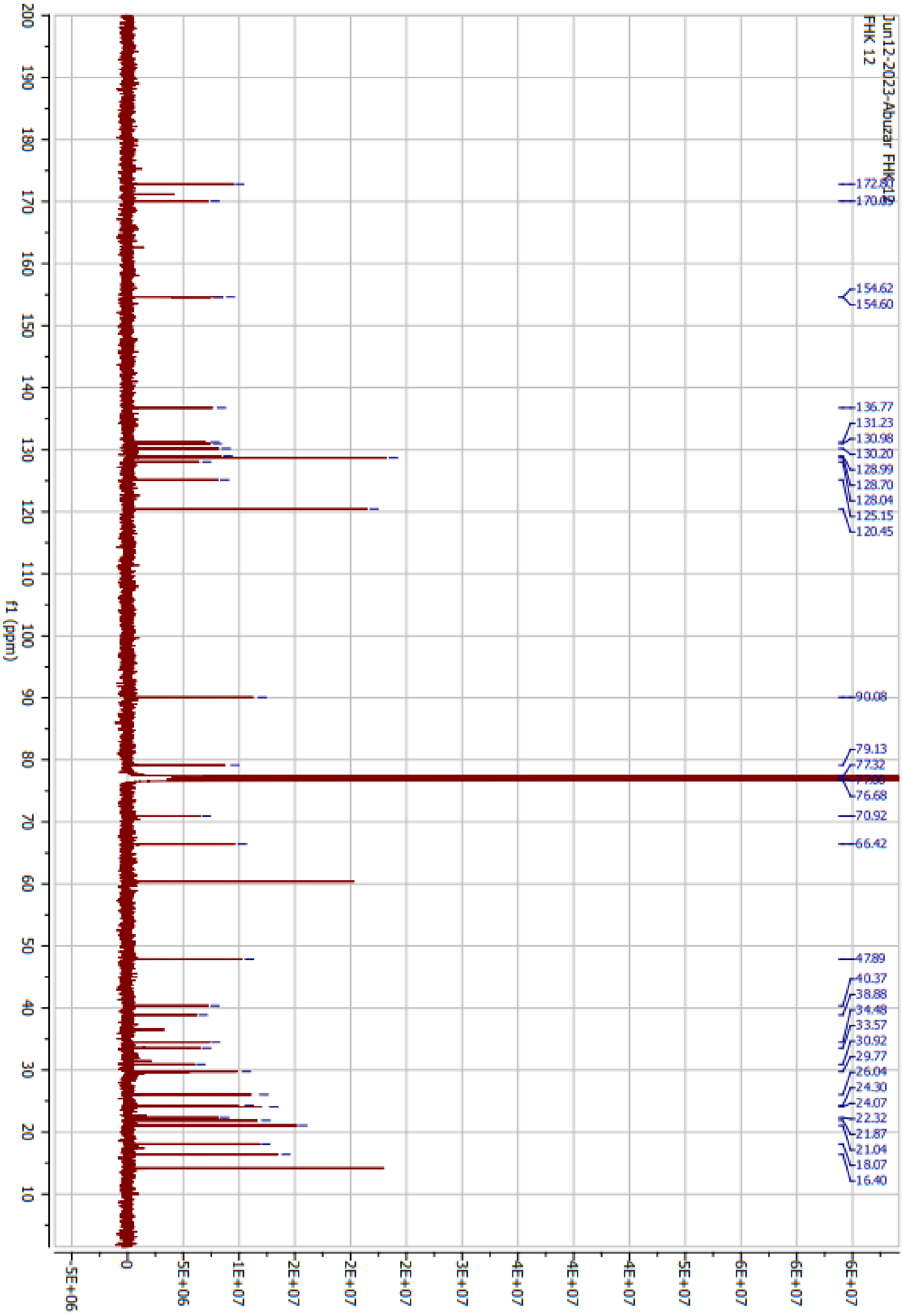

13C NMR for compound **5e**

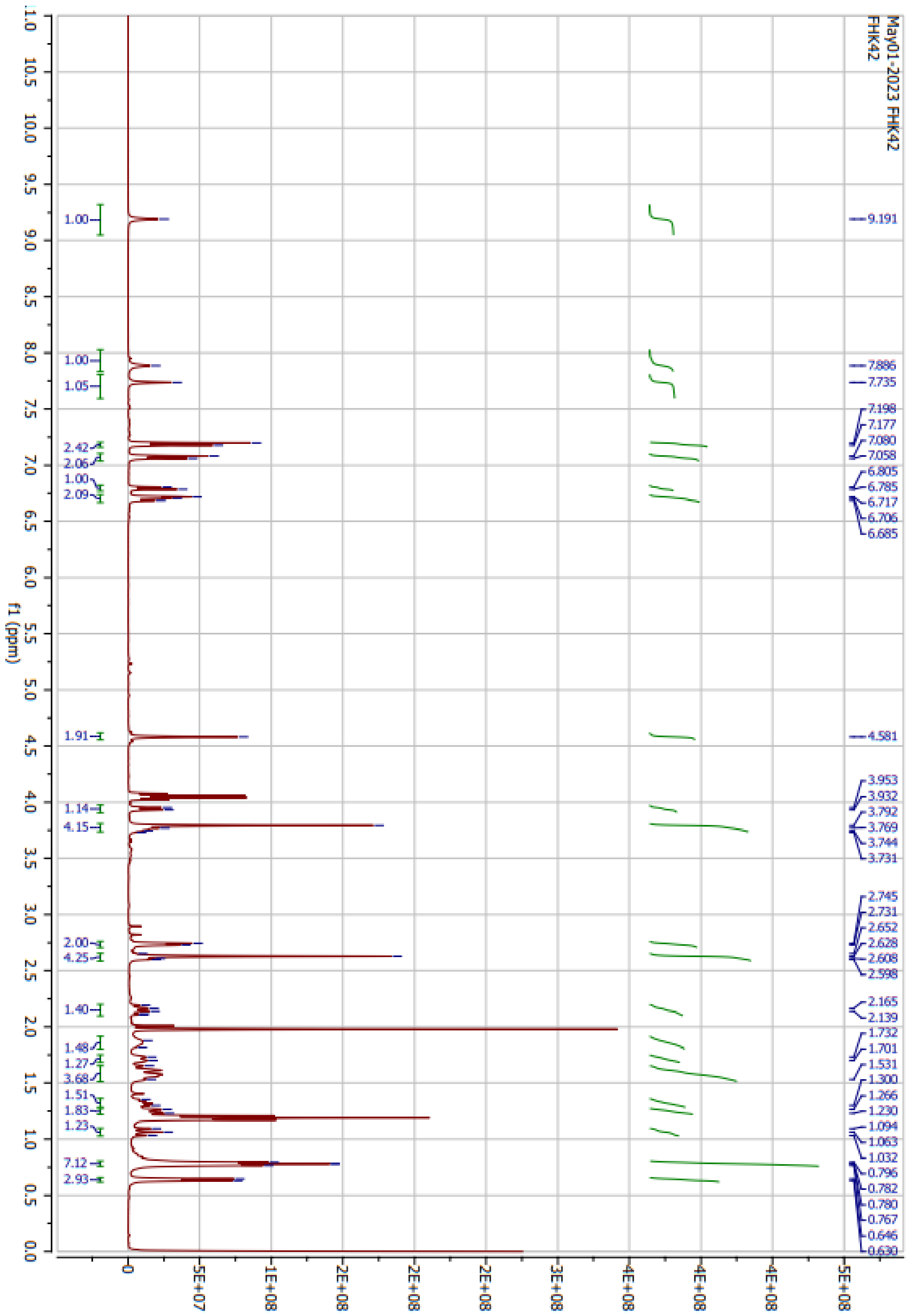

1H NMR for compound **5f**

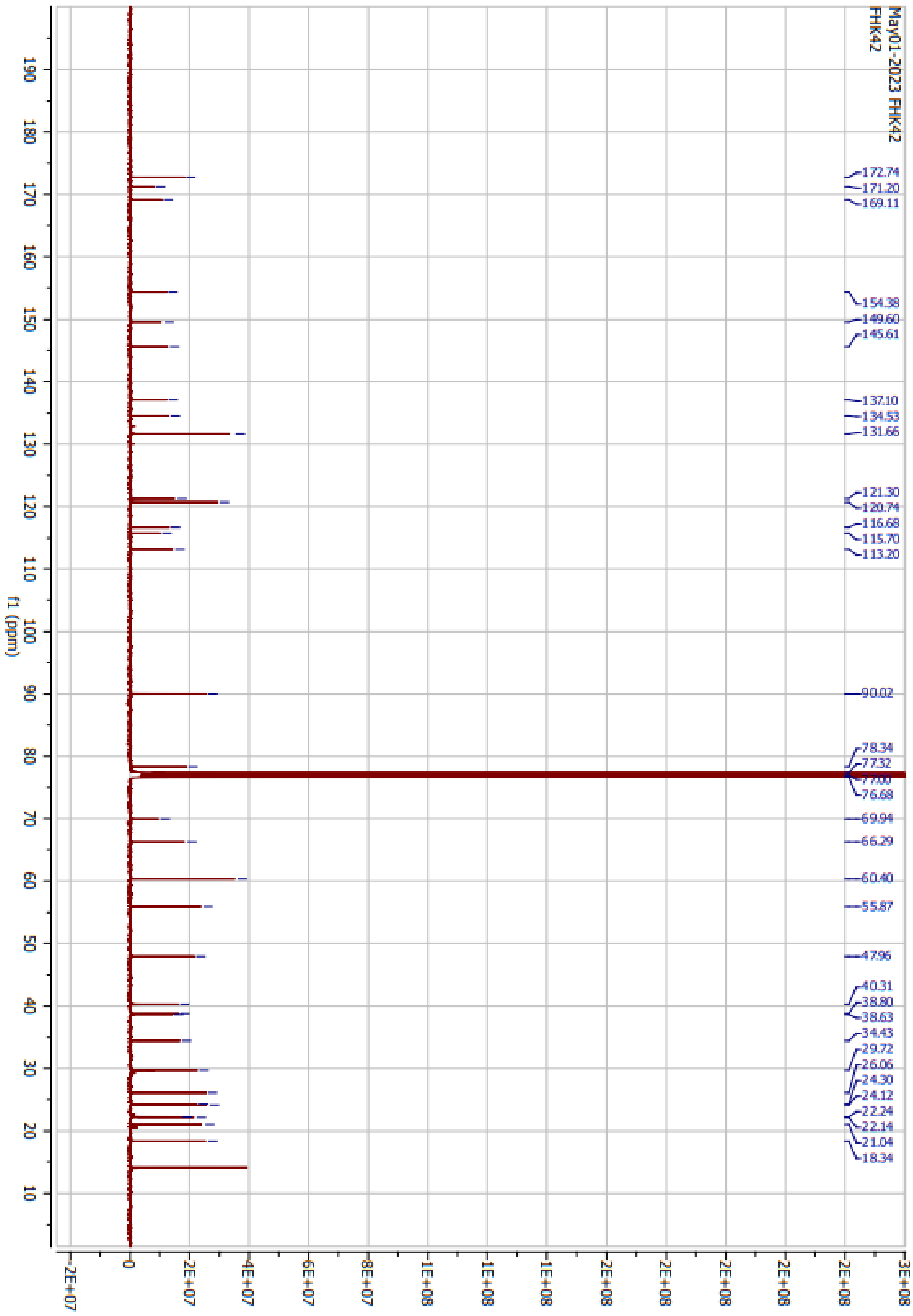

13C NMR for compound **5f**

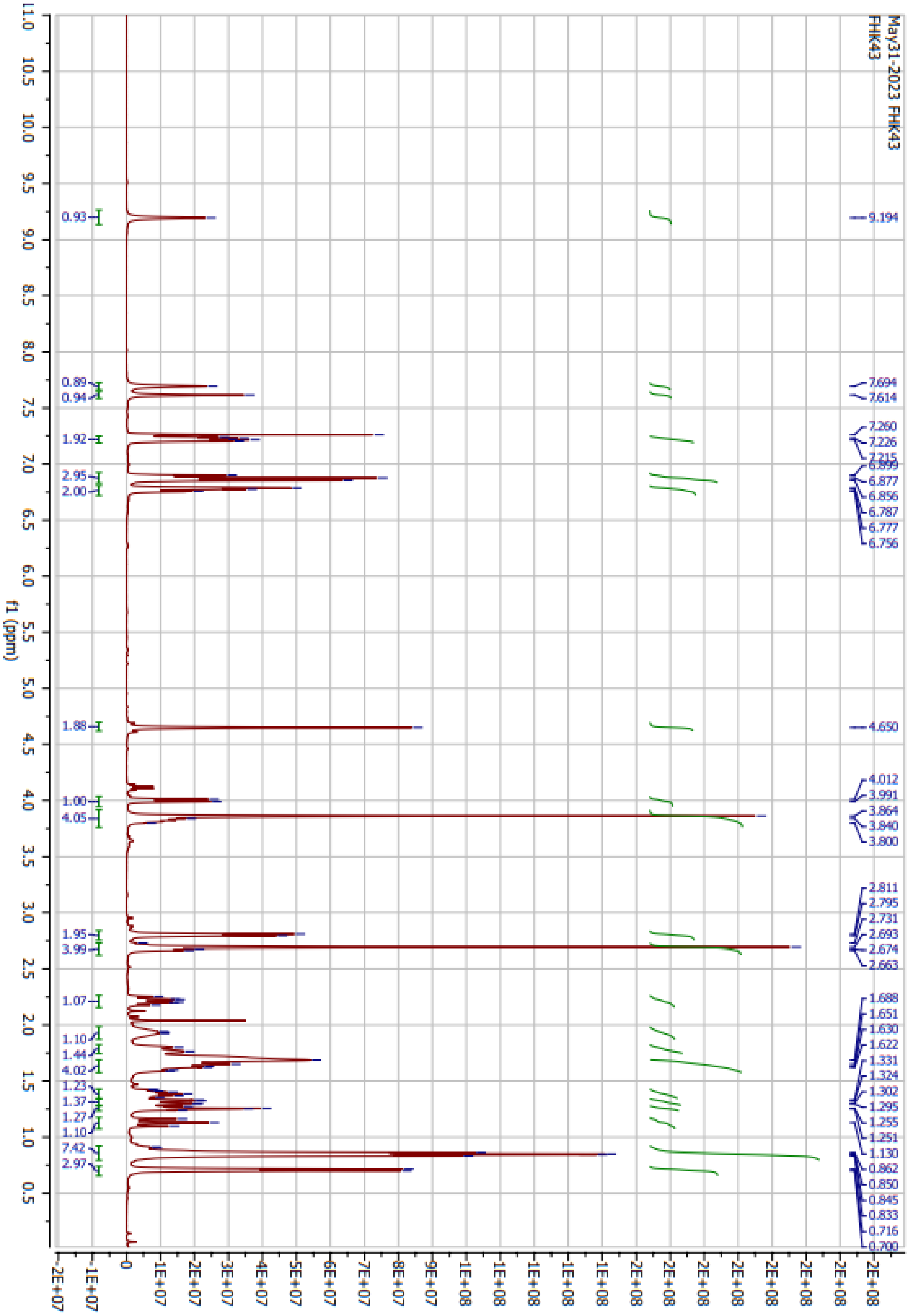

1H NMR for compound **5g**

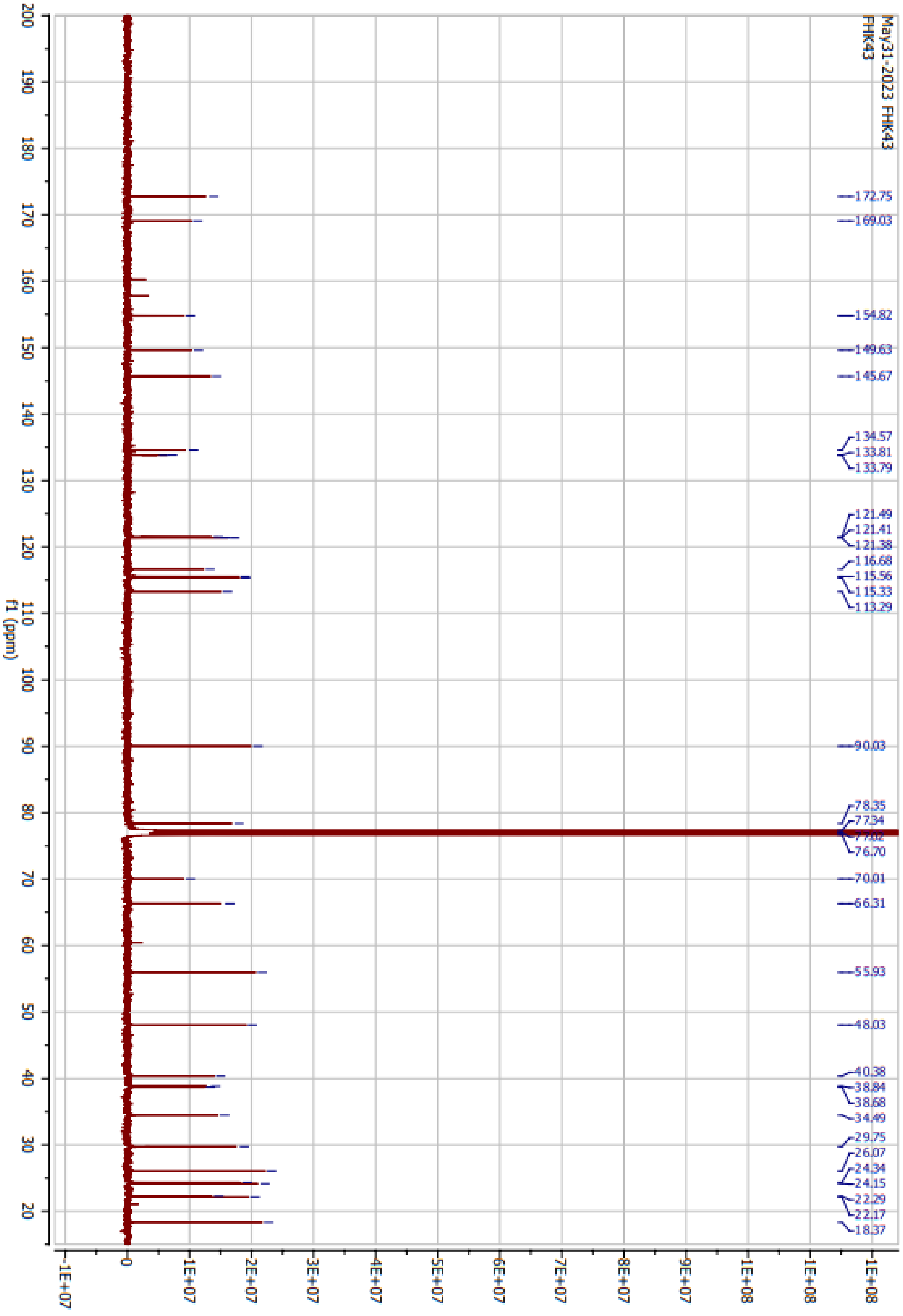

13C NMR for compound **5g**
